# Altered serotonin physiology in human breast cancers favors paradoxical growth and cell survival

**DOI:** 10.1186/bcr2448

**Published:** 2009-11-10

**Authors:** Vaibhav P Pai, Aaron M Marshall, Laura L Hernandez, Arthur R Buckley, Nelson D Horseman

**Affiliations:** 1 Department of Molecular and Cellular Physiology, University of Cincinnati, 231 Albert Sabin Way, Cincinnati, OH, 45267-0576, USA; 2 Systems Biology and Physiology Program, University of Cincinnati, 231 Albert Sabin Way, Cincinnati, OH, 45267-0576, USA; 3 James L. Winkle College of Pharmacy, University of Cincinnati, 3225 Eden Ave, Cincinnati, OH, 45267-0004, USA; 4 Current address: Tufts Center for Regenerative and Developmental Biology and Biology Department, Tufts University, 200 Boston Ave, Medford, MA, 02155, USA

## Abstract

**Introduction:**

The breast microenvironment can either retard or accelerate the events associated with progression of latent cancers. However, the actions of local physiological mediators in the context of breast cancers are poorly understood. Serotonin (5-HT) is a critical local regulator of epithelial homeostasis in the breast and other organs. Herein, we report complex alterations in the intrinsic mammary gland serotonin system of human breast cancers.

**Methods:**

Serotonin biosynthetic capacity was analyzed in human breast tumor tissue microarrays using immunohistochemistry for tryptophan hydroxylase 1 (TPH1). Serotonin receptors (5-HT1-7) were analyzed in human breast tumors using the Oncomine database. Serotonin receptor expression, signal transduction, and 5-HT effects on breast cancer cell phenotype were compared in non-transformed and transformed human breast cells.

**Results:**

In the context of the normal mammary gland, 5-HT acts as a physiological regulator of lactation and involution, in part by favoring growth arrest and cell death. This tightly regulated 5-HT system is subverted in multiple ways in human breast cancers. Specifically, TPH1 expression undergoes a non-linear change during progression, with increased expression during malignant progression. Correspondingly, the tightly regulated pattern of 5-HT receptors becomes dysregulated in human breast cancer cells, resulting in both ectopic expression of some isoforms and suppression of others. The receptor expression change is accompanied by altered downstream signaling of 5-HT receptors in human breast cancer cells, resulting in resistance to 5-HT-induced apoptosis, and stimulated proliferation.

**Conclusions:**

Our data constitutes the first report of direct involvement of 5-HT in human breast cancer. Increased 5-HT biosynthetic capacity accompanied by multiple changes in 5-HT receptor expression and signaling favor malignant progression of human breast cancer cells (for example, stimulated proliferation, inappropriate cell survival). This occurs through uncoupling of serotonin from the homeostatic regulatory mechanisms of the normal mammary epithelium. The findings open a new avenue for identification of diagnostic and prognostic markers, and valuable new therapeutic targets for managing breast cancer.

## Introduction

Evolution in cancers is a convergent phenomenon, during which heterogeneous genetic and epigenetic changes lead to similar ultimate tumor phenotypes. The essential phenotype of epithelial cancers (*e.g*., breast, liver, pancreas, prostate, and so on) includes: self-sufficiency for growth signals, insensitivity to growth inhibition, evasion of programmed cell death, apparently limitless replicative potential, sustained angiogenesis, and tissue invasion [1-4]. The convergent evolution of cancer phenotypes presents numerous prevention and treatment challenges because of the ability of cancer cells to exploit a variety of normal physiological processes out of context. In this study, we report extensive modifications of the recently-discovered mammary serotonin (5-hydroxytryptamine, 5-HT) system [5-9] in human breast cancers. This is the first account illustrating direct involvement of 5-HT in breast cancers, and shows that an important homeostatic signal is subverted by cancer cells, yielding paradoxical effects on growth and apoptosis.

Serotonin is a monoamine hormone and neurotransmitter that has been evolutionarily conserved, with functions stretching across the animal and plant phyla [10]. Although parochially known and studied as a neurotransmitter, with critical cognitive and behavioral functions in humans, 5-HT has numerous important peripheral functions in the gut, vasculature, immune system, and at wound sites [11-16]. Serotonin is synthesized in a two-step reaction from the amino acid L-tryptophan. The first and rate-limiting step in 5-HT synthesis is catalyzed by tryptophan hydroxylase (TPH), which is represented by neuronal (TPH2) and non-neuronal (TPH1) isoforms. Serotonin exerts its actions through a repertoire of greater than 15 different receptor proteins, belonging to seven discreet families. Six of the families of 5-HT receptors are G-protein-coupled, including G_i_: 5-HT_1_, G_s_: 5-HT_4,6,7_, and G_q/11_: 5-HT_2,5_. 5-HT_3 _is uniquely a ligand-gated cation channel, related to the nicotinic acetylcholine receptor. Another major player within the 5-HT system is the 5-HT reuptake transporter (SERT), which is involved in the uptake and clearance of extracellular 5-HT.

Mammary epithelial homeostatic mechanisms ensure normal tissue function during dramatic changes associated with pregnancy, lactation and involution. Serotonin is an integral part of this epithelial homeostatic system. In part, breast cancers arise through dysregulation of epithelial homeostatic systems [17,18].

Regulation of epithelial homeostasis by 5-HT is not exclusive to the mammary epithelium. Serotonin has been implicated in epithelial homeostasis of the lung, pancreas, liver and prostate. Moreover, dysregulation of 5-HT systems in these epithelia are associated with various pathologies, including cancer progression [19-29]. Consequently, alteration of local 5-HT signaling may be a common feature of cancer progression in epithelial tumors.

In these studies, we present the first analysis of the mammary 5-HT system in human breast cancer. Our results show extensive 5-HT signal modifications contributing to the cancer phenotype. These results provide a new theoretical framework for studying 5-HT signaling in a variety of epithelial cancers.

## Materials and methods

### Subjects

Primary human mammary epithelial cells (pHMEC), obtained from reduction mammoplasty under Institutional Review Board approval, were a generous gift from Eric R. Hugo at The University of Cincinnati; cell lines and anonymous tissue microarray specimens purchased from the National Cancer Institute were considered to be exempt. The research carried out in this article is in compliance with the Declaration of Helsinki.

### Cells and media

Primary human mammary epithelial cells (pHMEC) were prepared using a modification of previously described protocol [30]. Briefly, excised human mammary tissue was finely minced, transferred to conical tubes, and digested overnight at 37°C in M199 media containing 2.5 mg/ml BSA (Sigma-Aldrich, St. Louis, MO, USA), 0.1% collagenase type III (Worthington Biochemical Corporation, Lakewood, NJ, USA) and antibiotic-antimycotic (Invitrogen, Carlsbad, CA, USA). Digested tissue was pelleted by centrifugation, washed in phosphate buffered saline (PBS), and either plated in the pHMEC media (see below) or frozen back for later use.

All the cell lines used in the aforementioned studies were procured from the American Type Culture Collection (ATCC), and used within 10 passages after acquisition. MCF10A media consisted of the following: DMEM-F12 50:50 (Invitrogen) supplemented with 5% horse serum, 2 mM L-glutamine, 10 μg/ml insulin (Sigma-Aldrich), 20 ng/ml EGF (Upstate Biotechnology, Waltham, MA, USA) 0.5 μg/ml hydrocortisone and antibiotic/antimycotic (Invitrogen). pHMEC media contained DMEM-F12 50:50, 5% FBS (Hyclone, Logan, UT, USA), insulin (Sigma-Aldrich), hydrocortisone (Sigma-Aldrich), EGF (Upstate), 1 ng/ml cholera toxin (Sigma-Aldrich) (except where noted) and antibiotic-antimycotic (Invitrogen). MDA-MB-231 cells were cultured in DMEM (Invitrogen) supplemented with 10% FBS, L-glutamine and antibiotic-antimycotic (Invitrogen). T47D cells were grown in same media as MDA-MB-231 with the addition of 5 μg/ml of insulin. MCF7 cells were grown in media consisting of DMEM-F12 50:50 (Invitrogen) supplemented with 10% FBS, L-glutamine, 1 mM sodium pyruvate (Invitrogen), 1× concentration of non-essential amino acids (Invitrogen), and antibiotic-antimycotic (Invitrogen).

### Immunohistochemistry and immunofluroscence

Paraformaldehyde fixed paraffin embedded mouse mammary gland sections and tissue microarray sections were deparaffinzed in xylene and rehydrated in decreasing concentrations of ethanol from 100% to 50%. Endogenous peroxidases were blocked by incubation in 3% H_2_O_2 _at room temperature for 30 min. Antigen retrieval was performed using borate buffer pH = 8.5 (80 mM boric acid, 20 mM sodium borate) in a microwave (60% power) twice for 5 min. Sections were then permeabilized using 0.2% Triton X-100 (Sigma-Aldrich) in PBS for 30 min followed by normal serum blocking for 1 h at room temperature and incubation in primary antibody (sheep anti-TPH 1:100 Abcam, Cambridge, MA, USA) overnight at 4°C in a humid chamber. The immune reaction was visualized using HRP-conjugated secondary antibody (Sigma-Aldrich) and ABC-DAB system (Zymed, S. San Francisco, CA, USA; and Vector Labs, Burlingame, CA, USA, respectively).

Cells grown on coverslips were fixed in 4% paraformaldehyde. Cells were permeabilized in 0.1% Triton X-100, incubated in borate buffer overnight at 75°C and incubated in primary antibodies overnight at 4°C. Images were collected using a Zeiss LSM510 Confocal Microscope, Göttingen, Germany) using the Zeiss LSM Image Software version 3.5, Munich, Germany)

### RT-PCR and Western blot

Total cellular RNA was extracted using TRI-REAGENT (Molecular Research Center, Inc., Cincinnati, OH, USA) according to the manufacturer's instructions. Two μg of RNA was subjected to reverse transcription by standard methods. One μl cDNA was used for 25 μl PCR reactions. For primer information, please see Table S1 in Additional data file 1.

Cellular protein extracts were prepared using the Cell Lysis kit (Cell Signaling Technology, Boston, MA, USA) as per the manufacturer's instructions. Proteins were quantified using Lowry assay and equal amounts were separated on SDS-PAGE gel. After transferring the proteins to nitrocellulose membrane, the specific proteins were visualized using specific antibodies and detected using HRP tagged secondary antibodies.

### Proliferation assay and Trypan blue assay

After experimental treatments, cell proliferation was measured by colorimetric assay based on cleavage of a tetrazolium salt 3-(4,5-dimethylthiazol-2-yl)-5-(3-carboxymethoxyphenyl)-2-(4-sulfophenyl)-2H-tetrazolium (MTS) by mitochondrial dehydrogenase enzyme present in proliferating cells (Cell titer 96 - Promega, Madison, WI, USA). For trypan blue experimental procedures, the cells were gently trypsinized and re-suspended in 0.2% trypan blue solution and counted using a hemocytometer.

### Tissue microarray

Human breast cancer tissue microarrays were purchased from The National Cancer Institute (Bethesda, MD, USA [31]. The array has 288 cores in quadruplicate with tissue matched controls. A modified histochemical-score (H-score) [32,33] system was used to evaluate the breast cancer tissue microarrays. An H-score comprises of a semi-quantitative assessment of both the intensity of staining and percentage of positive mammary epithelial cells. For intensity, a score of 0 to 3, corresponding to negative, weak, positive and strong positive was recorded blindly by two independent observers with final scores resulting in an average. It was not necessary to correct the staining intensities to account for the percentage of positive cells because of uniform epithelial staining within given specimens.

### Statistics

Each experiment in cultured cells and tissues was replicated three or four times, and representative results are shown in figures. Differences in means were tested by ANOVA with Tukey's post-hoc test for multiple groups, and Student's T-test or an equivalent non parametric test (Mann-Whitney U test) for comparisons of two means. Significance was accepted for *P *< 0.05.

## Results

### Elevated TPH1 in human breast cancer cells

**Figure 1:**
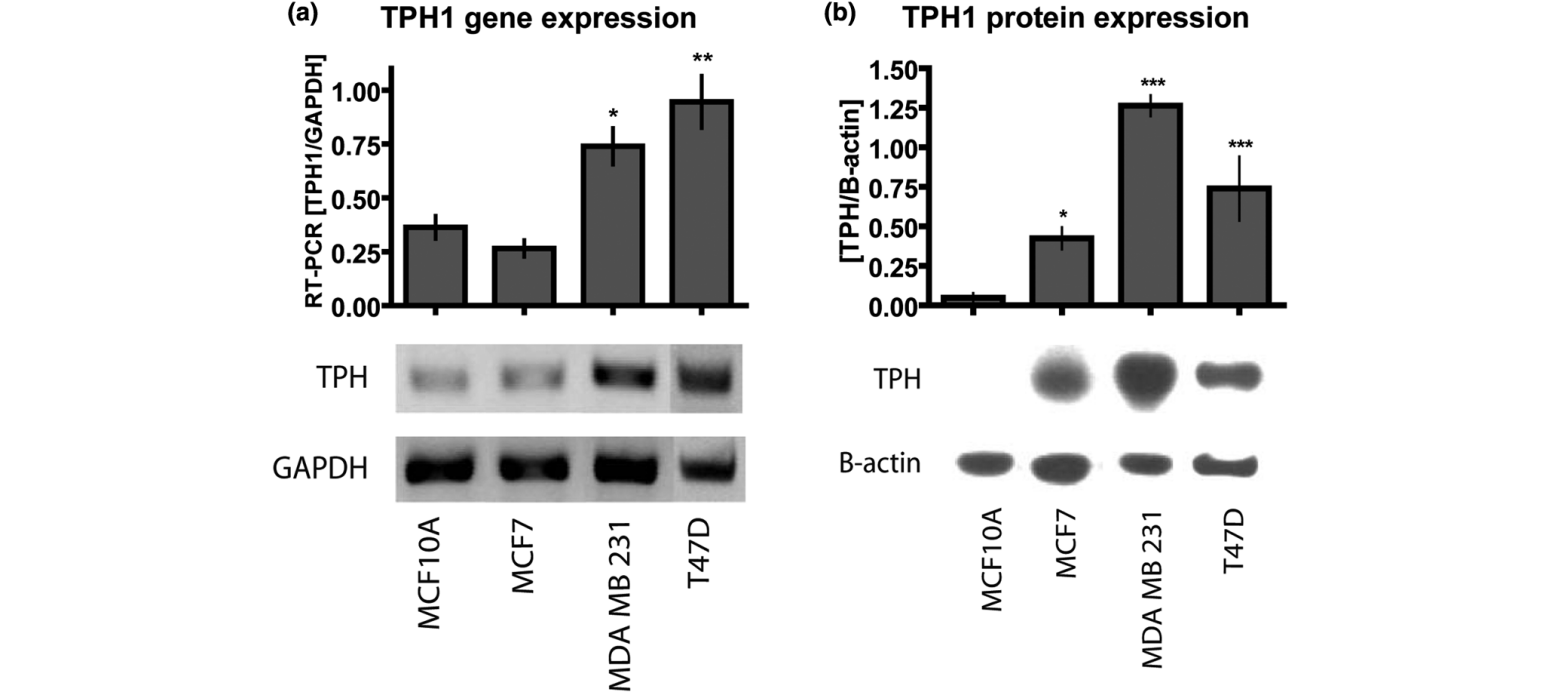
TPH1 expression in non-transformed and breast cancer cells. **(A) **Bar graphs representing TPH1 mRNA levels in indicated cell lines as detected by semi-quantitative RT - PCR reactions and normalized to GAPDH. Shown below is a representative picture of resulting PCR reactions. **(B) **Bar graphs and electophoretic bands showing TPH1 protein levels in the extracts of indicated cells normalized to β-actin. Error bars in (A) and (B) represent ± SEM. *P < 0.05, **P < 0.01 and ***P < 0.001 (one-way ANOVA) in comparison to MCF10A cells.

TPH1 catalyzes the first and the rate-limiting step in 5-HT biosynthesis. We have previously shown that TPH1 is expressed in the mammary gland and TPH1 expression directly correlates with 5-HT production [5,6]. We therefore addressed the question of whether there were any changes in 5-HT synthetic capacity of breast cancer cells. Toward this end, we analyzed TPH1 mRNA levels by reverse-transcriptase-couple polymerase chain reaction (RT-PCR) in different human breast cancer cells (MCF7, MDA-MB-231 and T47D), and compared them to that of non-transformed human mammary epithelial cells (MCF10A). The mRNA levels of TPH1 were elevated more than two-fold in MDA-MB-231 and T47D cells compared to that of MCF10A cells (Figure 1A). The TPH1 protein was analyzed by western blot in extracts of these cells and was significantly elevated not only in MDA-MB-231 and T47D, but also in MCF7 cells, demonstrating that 5-HT biosynthetic capacity was increased in all breast cancer cell lines tested, with the highest level of expression occurring in MDA-MB-231 cells (Figure 1B). This was contrary to what occurred in MCF10A cells, where TPH1 protein was near the lower limit of detection by western blotting (Figure 1B). An additional factor that regulates cellular exposure to 5-HT is SERT, which pumps 5-HT back into the cells contributing to the recycling and controlling the extracellular concentration of 5-HT. We assessed SERT protein levels and found them to be similar among these cell lines [see Figure S1 in Additional data file 1].

### Nonlinear association of TPH1 with breast cancer progression

**Figure 2:**
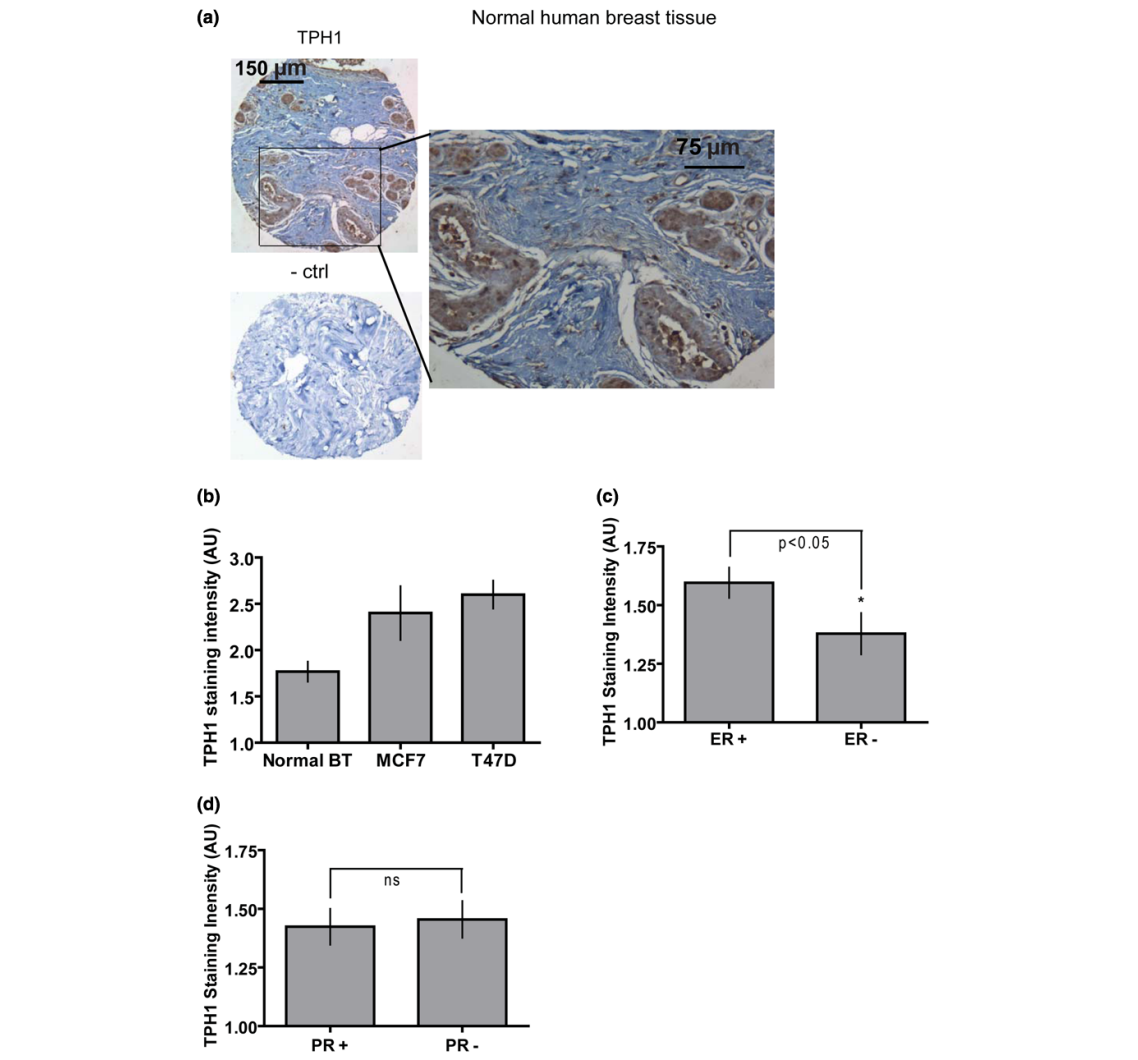
Changes in TPH1 signal in human breast tumors. **(A) **Representative image of normal human breast tissue (BT) from the tissue microarray stained for TPH1 (brown) and counterstained with hematoxylin. A corresponding no primary antibody control section is depicted on the bottom left. Magnified image shows details of epithelial staining and occasional positive cells in stroma. **(B-D) **Results depicting quantification of TPH1 immunostaining from blindly scored tissue microarray sections. The section scores for TPH1 were sorted as per human cancer cell line core (B), estrogen receptor (ER) status (C) and progesterone receptor (PR) status (D). TPH1 staining was reduced in ER negative tumors, but unaffected by PR status. Error bars represent ± SEM. *P < 0.05 in comparison with normal BT for (A).

To gain insight into the possible association between altered 5-HT synthesis and breast cancer progression in actual human tumors, we analyzed TPH1 in histological specimens. The specimens used were human breast cancer tissue microarrays acquired from The National Cancer Institute Cooperative Breast Cancer Tissue Resource [31]. Quadruplicate samples of 288 cores comprised the array, which included both tissue-matched non-cancer and non-diseased controls, along with cell line controls. Figure 2A shows a representative section of normal human breast tissue from an array at two different magnifications, stained for TPH1. In normal breast tissue the epithelium was dispersed in an orderly fashion within the stroma, and the epithelium stained distinctly for TPH1. Scattered blood vessel-associated cells were also positive for TPH1 within the stroma. A modified H-score [34,35] was used by blinded examiners to score the stained tissues. Sections were scored on a scale of 0 to 3 with respect to TPH1 staining separately within epithelia and stroma, using a key generated from among the array sections [see Figure S2 in Additional data file 1]. Cores of cell lines on the array (MCF7 and T47D) validated the scoring for TPH1. Similar to the expression pattern we had seen previously in the cell lines (Figure 1), TPH1 staining intensity was significantly elevated in the cancer cell lines, compared with normal breast tissue (Figure 2B).

The tissue specimens on the array were all from primary tumor sites, and were scored based on a variety of pathological criteria. An obvious and clinically meaningful characterization is reproductive steroid receptor (estrogen receptor (ER) and progesterone receptor (PR)) status. Levels of TPH1 were lower in ER negative cases, compared with ER positive cases. There was no difference in TPH1 levels associated with PR status of the breast cancers (Figure 2C and 2D, respectively).

**Figure 3:**
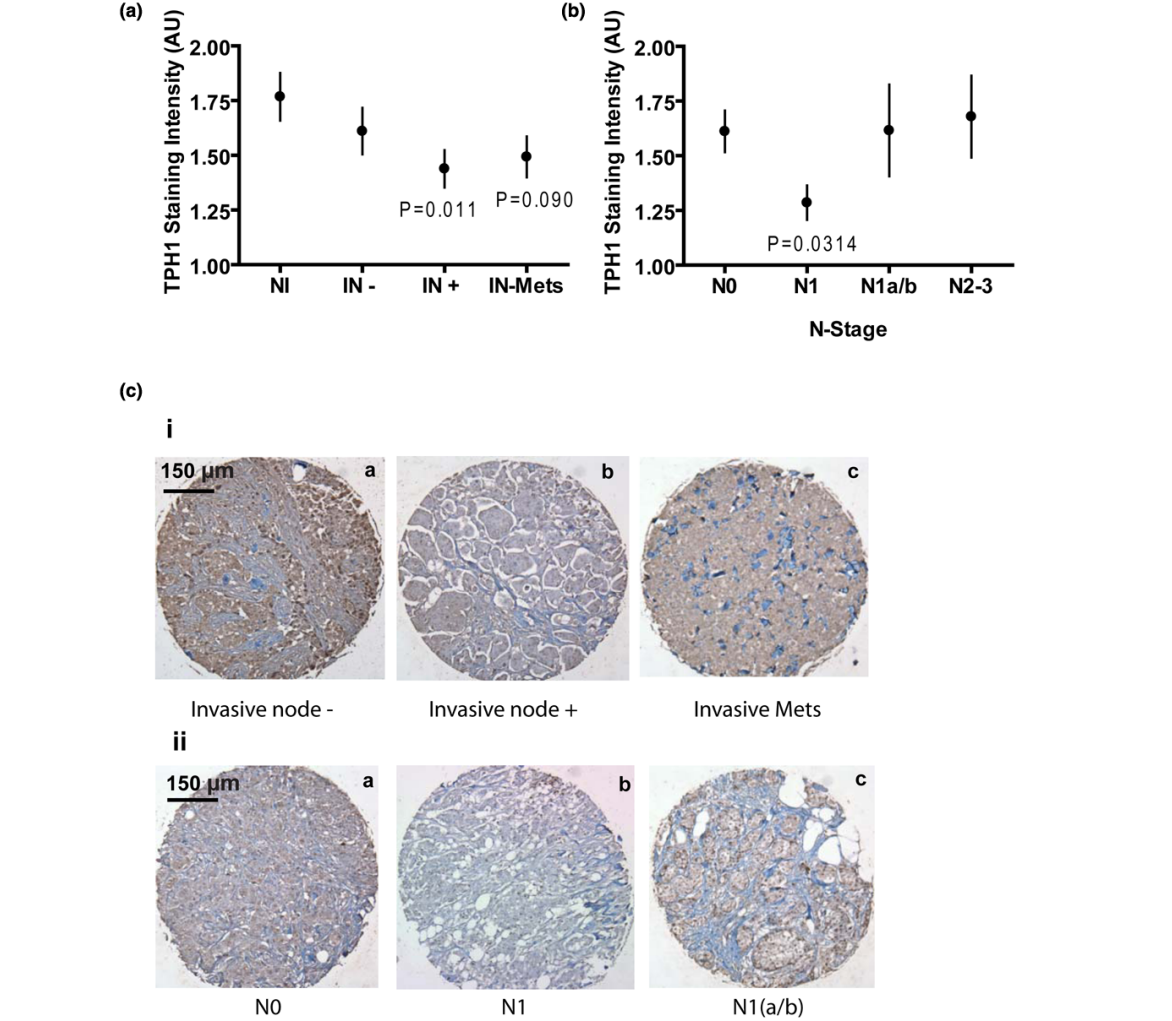
Nonlinear association between TPH1 and cancer progression. **(A-B) **Results depicting quantification of TPH1 immunostaining from blindly scored tissue microarray sections. Data were sorted based on the indicated criteria. Data were analyzed by one-way ANOVA with *P*-values as noted in comparison with NI for (A) and N0 for (B). Error bars represent ± SEM. (A) TPH1 scores in samples identified as non-invasive (NI); invasive, node-negative (IN-); invasive, node-positive (IN+); and invasive with distant metastases (IN-Mets). (B) Human breast cancer tissue sections scored for TPH1 were sorted as per different stages of nodal metastasis (N-stage: N0, no regional lymph node involvement; N1, Mets to movable ipsilateral nodes; N1a/b, Mets up to 20 mm to 4 or more nodes with extension beyond node capsule; N2-3, Mets to ipsilateral nodes that are fixed to one another or to other structures and to internal mammary lymph nodes). **(C) **Representative microarray sections stained for TPH1 from sorted groups as indicated below each image. Brown/red dye indicates TPH1 stain, blue is hematoxylin.

Segregating the cases crudely according to tumor size and according to T-stage (another size-based classification) showed an inverse relationship between tumor size (>20 mm) and TPH1 staining [see Figure S3 in Additional data file 1]. When the breast cancer cases were sorted according to invasion and progression criteria, TPH1 staining showed a set of nonlinear relationships (Figure 3). Staining for TPH1 was lower in locally-invasive (IN+) cases compared with non-invasive samples (NI), which included both normal tissue and ductal carcinoma *in situ *(DCIS). However, TPH1 was high in cases of tumors with distant metastases (IN-Mets) (Figure 3A). The relationship of TPH1 staining to progression was clearest among samples sorted according to N-stage (extent of lymph node involvement) (Figure 3B). Staining for TPH1 was significantly decreased in the N1 staged tumors (one to three ipsilateral nodes). However, with progression to N1a/b stage (micrometastases to four or more nodes, including extension beyond the node capsule) and higher stages (N2/3), the TPH1 staining was elevated. Representative core sections from each sorted category are depicted in Figure 3C.

### 5-HT receptor expression in association with breast cancer progression

**Figure 4:**
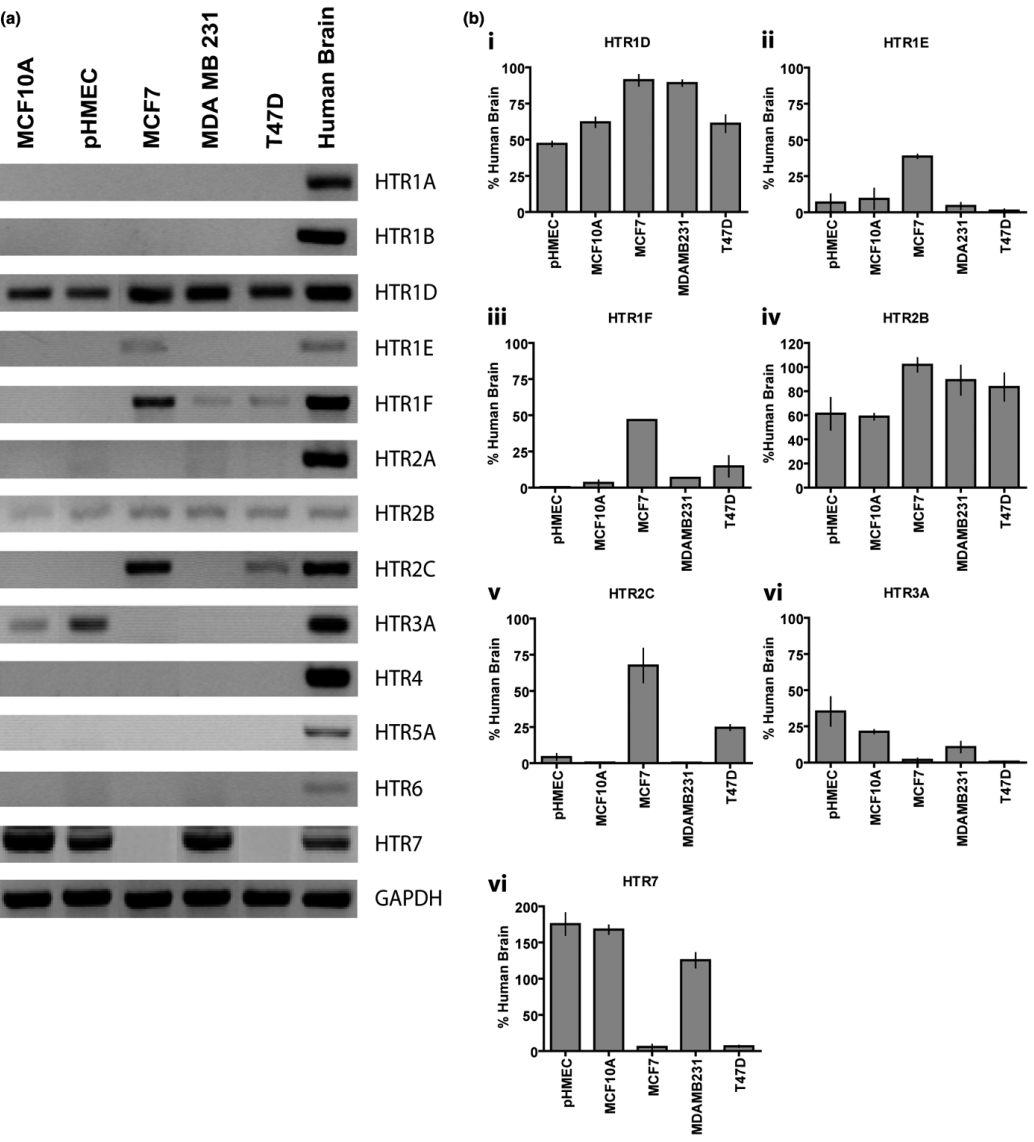
Difference in 5-HT receptor isoform gene expression between non-transformed (pHMECs and MCF10A) and breast cancer cells (MCF7, MDA-MB-231 and T47D). **(A) **Representative RT-PCR reactions (mRNA transcripts) for indicated 5-HT receptors, in cells as indicated at the top. Human brain (hypothalamus) is used as a reference sample for each receptor. For primer information please see Table S1 in Additional data file 1. **(B) **Densitometric quantification (semi-quantitative) of transcript levels relative to human brain reference samples (100%). Each column represents average with error bars representing ± SEM of at least three independent reactions.

To determine the expression of 5-HT receptor mRNA profiles, we performed a comprehensive analysis by RT-PCR (Figure 4). In addition to HTR7, which has been studied in detail in the breast [6,7], we report for the first time the expression of HTR1D, 2B and 3A in an untransformed cell line and primary human mammary epithelial cells (MCF10A and pHMEC) (Figure 4). The HTR1D and 2B were also present in all breast cancer cells tested and, using human hypothalamus as the reference level, expression in the cancer cells was elevated compared to the untransformed cells (Figure 4Bi and 4Biv).

Among the receptor isoforms that were expressed differentially in breast cancer cell lines, HTR3A was markedly downregulated in all of the cancer lines (Figure 4A and 4Bvi). Three receptor types were upregulated in MCF7 to levels that were at least 40% of the brain (1E, 1F, 2C) (Figure 4A, Bii, iii and 4Bv). MDA-MB-231 expressed 1F, and T-47D expressed both 1F and 2C at modest levels relative to the brain (Figure 4B). No detectable transcripts for HTR1A, 1B, 2A, 4, 5A and 6 were found in the untransformed (MCF10A and pHMECs) or breast cancer cells (MCF7, T47D and MDA-MB-231) (Figure 4A) and [see Figure S4 in Additional data file 1]. HTR7 was expressed in MCF10A [6,7], pHMEC and MDA-MB-231 cells. In contrast, MCF7 and T47D, both of which are ER positive cancer cells, lacked any detectable HTR7 transcript (Figure 4A).

To address the question of 5-HT receptor profiles in breast cancers, we examined expression using the *Oncomine *database [36,37] to search preliminarily for patterns of 5-HT receptor gene expression (Table 1). Among all of the receptor isoforms, HTR1B, 1D, 1F, 2A, 2B, 2C, 3, 4, 5A, 7 were found to be expressed in breast tissues (cancer and other). Of these receptors, the expression levels of HTR1D, 2A and 3 were unchanged in the archived studies (data not shown). The HTR1D pattern was in accord with the breast cancer cell lines (Table 1). HTR1B and 2A were not expressed in either pHMEC or established breast cell lines (Figure 4), which implies that expression in tumor specimens represents the presence of stromal or vascular elements, which typically express HTR1B and 2A in smooth muscle cells [38,39].

**Table 1:** Modification of 5-HT receptor expression with human breast cancer status - Oncomine study

Study Type	Receptor Type	Variable	Direction of Change	*P*-value (T-test)	Oncomine data source
**Tumor grade/stage**	HTR2B	Normal vs Breast Carcinoma	Higher in Cancer	0.002	Richardson *et. al*. [77]
		Tumor stage N0-N3	Increase with stage	0.006	Bittner *et. al*. [78]
	HTR2C	Tumor stage N0-N3	Increase with stage	0.009	Yu *et. al*. [79]
	HTR7	Grade I to III	Increase with grade	<0.0001	Ivshina *et. al*. [80]
				0.0001	Miller *et. al*. [81]
				<0.01	Ginestier C *et. al*. [82]
**More aggressive vs Less aggressive breast cancers**	HTR1F	5 yrs recurrence- vs 5 yrs recurrence+	Higher in recurring tumors	0.005	VantVeer *et. al*. [83]
	HTR2B	Lymph node- vs Lymph node+	Higher in lymph node +	0.006	Chin *et. al*. [84]
	HTR2C	Primary vs Metastatic tumors	Higher in metastatic	0.001	Bittner *et. al*. [78]
		Her2/neu- vs Her2/neu+	Higher in Her2/neu+	0.00057	Minn *et. al*. [85]
				0.002	Hess KR *et. al*. [86]
	HTR5A	P53-mutant vs p53-WT	Higher in p53-mutant	<0.0001	Miller *et. al*. [81]
	HTR7	P53-mutant vs p53-WT	Higher in p53-mutant	0.008	Miller *et. al*. [81]
**Steroid receptor status**	HTR1F	ER+ vs ER-	Higher in ER+	0.0001	Sotiriou *et. al*. [87]
	HTR2B	ER+ vs ER-	Higher in ER+	0.00014	Wang *et. al*. [88]
				0.0004	Sotitiou *et. al*. [87]
				0.00014	Desmedt *et. al*. [89]
		Luminal vs Basal Tumors	Lower in Basal (ER-)	<0.0001	Farmer *et. al*. [90]
	HTR4	ER+ vs ER-	Higher in ER+	0.001	Chi *et. al*. [84]
	HTR7	ER+ vs ER-	Higher in ER+	<0.0001	VanderVijer *et. al*. [91]
				0.00087	Bittner *et. al*. [78]
**Human Mammary Cell lines**	HTR2B	HMEC-Normal (GFP) vs c-Myc transformation	Higher in c-Myc transformed cells	0.001	Bild AH *et. al*. [92]
	HTR7	HMEC-Normal (GFP) vs activated H-Ras	Higher in H-Ras cells	0.00028	Bild AH *et. al*. [92]

Receptor mRNAs encoding 5-HT_1F _(HTR1F), 5-HT_2B _(HTR2B), 5-HT_2C _(HTR2C), 5-HT_4 _(HTR4), 5-HT_5A _(HTR5A) and 5-HT_7 _(HTR7) sorted as per tumor stage/grade, ER status, tumor aggressiveness and human mammary cell line transformation. Abbreviations: (ER+) - Estrogen Receptor Positive, (ER-) - Estrogen Receptor Negative. (HMEC) - human mammary epithelial cells.

A significant subset of 5-HT receptor mRNAs (HTR1F, 2B, 4 and 7), were significantly decreased in ER negative tumors, relative to ER positive tumors (Table 1). These observations were confirmed by multiple independent studies. HTR2B expression was also lower in basal tumors (ER negative), compared with luminal tumors, which are most commonly ER positive (Table 1).

Expression levels for HTR1F, 2B, 2C, 5A and 7 showed overall increases in breast cancers. HTR2B, which is expressed in untransformed human mammary epithelium (Figure 4), was elevated in carcinomas and was found to increase with tumor stage, and concomitantly was higher in lymph node-positive tumors as compared to node-negative tumors (Table 1). This observation was supported by a study of HMECs, showing c-Myc transformation induced an increase in HTR2B expression (Table 1). Similar to HTR2B, HTR2C showed an increase in expression with tumor stage. The HTR2C pattern was in accord with the increased expression observed in human breast cancer cell lines (Figure 4). Analogously, HTR2C expression was found to be higher in metastatic and Her2/neu-overexpressing tumors. The expression of HTR7 increased with tumor grade, and was higher in p53-mutated tumors (Table 1). This observation also was supported by a study of HMECs, showing H-Ras transformation induced increases in HTR7 expression. Other 5-HT receptor expression changes in breast cancers included HTR1F (higher in recurring tumors) and HTR5A (higher in p53-mutant) (Table 1).

The variety of examples showing aberrant expression of 5-HT receptors in breast cancers suggests that multiple modifications of 5-HT signaling can contribute to the loss of tissue homeostasis during tumor progression.

### 5-HT-induced apoptosis and morphological transition

**Figure 5:**
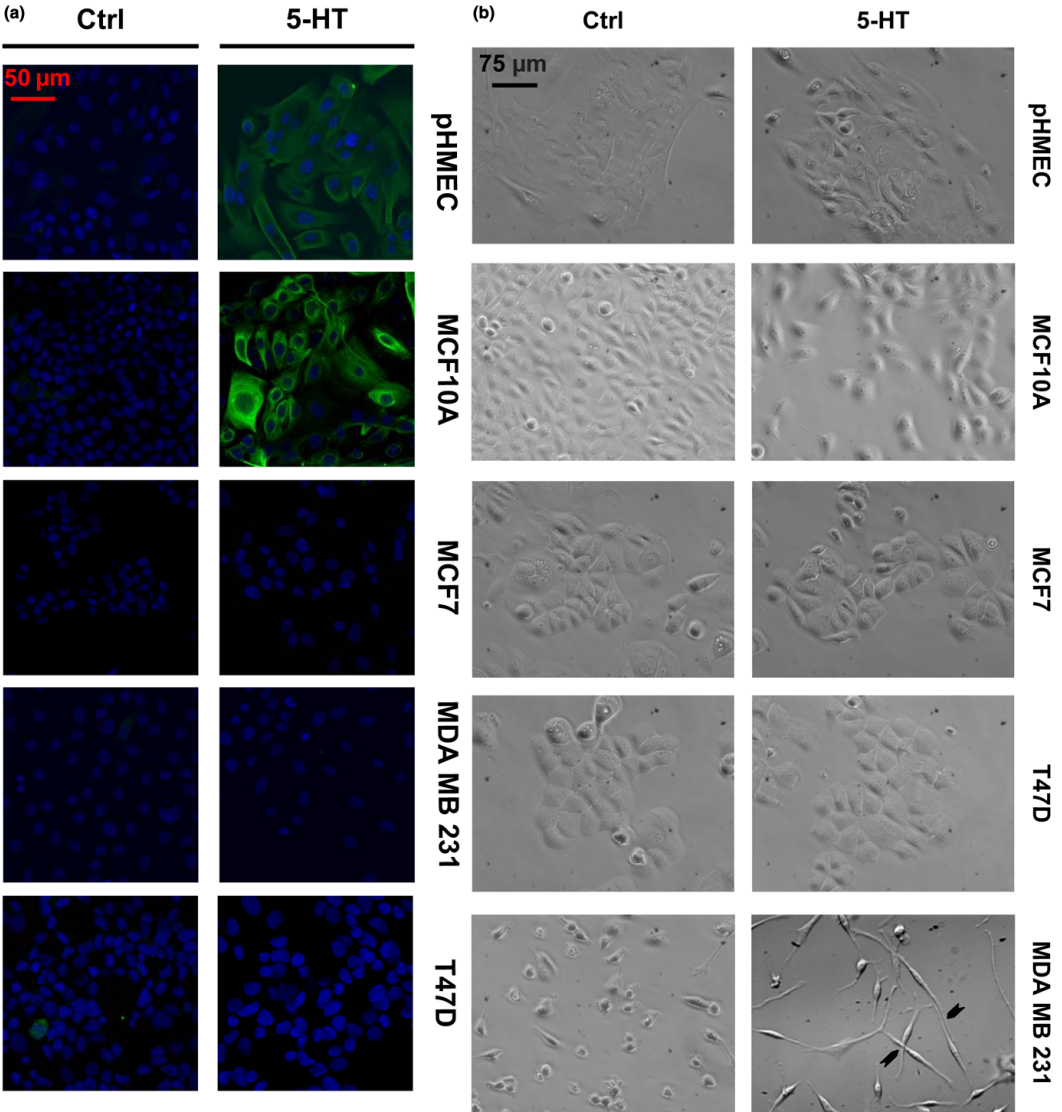
5-HT influence on apoptosis and morphological transition in breast cells. **(A) **Staining for apoptosis marker cleaved caspase-3 (green), counterstained for nuclei (blue) for the indicated cells with or without 5-HT (7.5 × 10^-4 ^M) treatment for 72 h in serum free media. **(B) **Phase-contrast images of the indicated cells with or without 5-HT (7.5 × 10^-4 ^M) treatment for 72 h. Arrowheads point to appendages at intersections between adjacent cells.

Our previous studies on 5-HT physiology in mammary gland cells revealed the critical roles of 5-HT in regulating epithelial homeostasis during involution, which is characterized by epithelial tissue regression [5-7,40,41]. One expected effect of elevated 5-HT activity in the normal breast is widespread apoptotic cell death. Hence, we tested the effects of 5-HT on apoptosis in mammary epithelial cells. The pHMEC and MCF10A cells, as expected, showed significant increases in active caspase 3 staining when treated with 5-HT, as compared to untreated controls (Figure 5A). In contrast, all of the breast cancer cell lines were highly resistant to 5-HT-induced apoptosis under similar experimental conditions.

In response to 5-HT, MDA-MB-231 cells underwent dramatic changes in their morphological phenotype, assuming exaggerated fibroblastic spindle morphologies (Figure 5B). In the presence of 5-HT, the MDA-MB-231 cells projected long axial appendages, which inconsistently contacted neighboring cells, sometimes crossing cells without apparent interactions. Previously, similar changes in morphology of MDA-MB-231 cells have been associated with exaggerated motility and invasiveness [42,43]. No similarly obvious phenotypic effects of 5-HT treatment were observed in MCF10A, pHMEC, T47D or MCF7 cells, suggesting that this response to 5-HT may be limited to the most aggressively-transformed breast cancer cells, which have undergone an epithelial-mesenchymal transition (Figure 5B).

### Serotonin inhibits growth of untransformed human mammary epithelial cells via the 5-HT_7 _receptor

**Figure 6:**
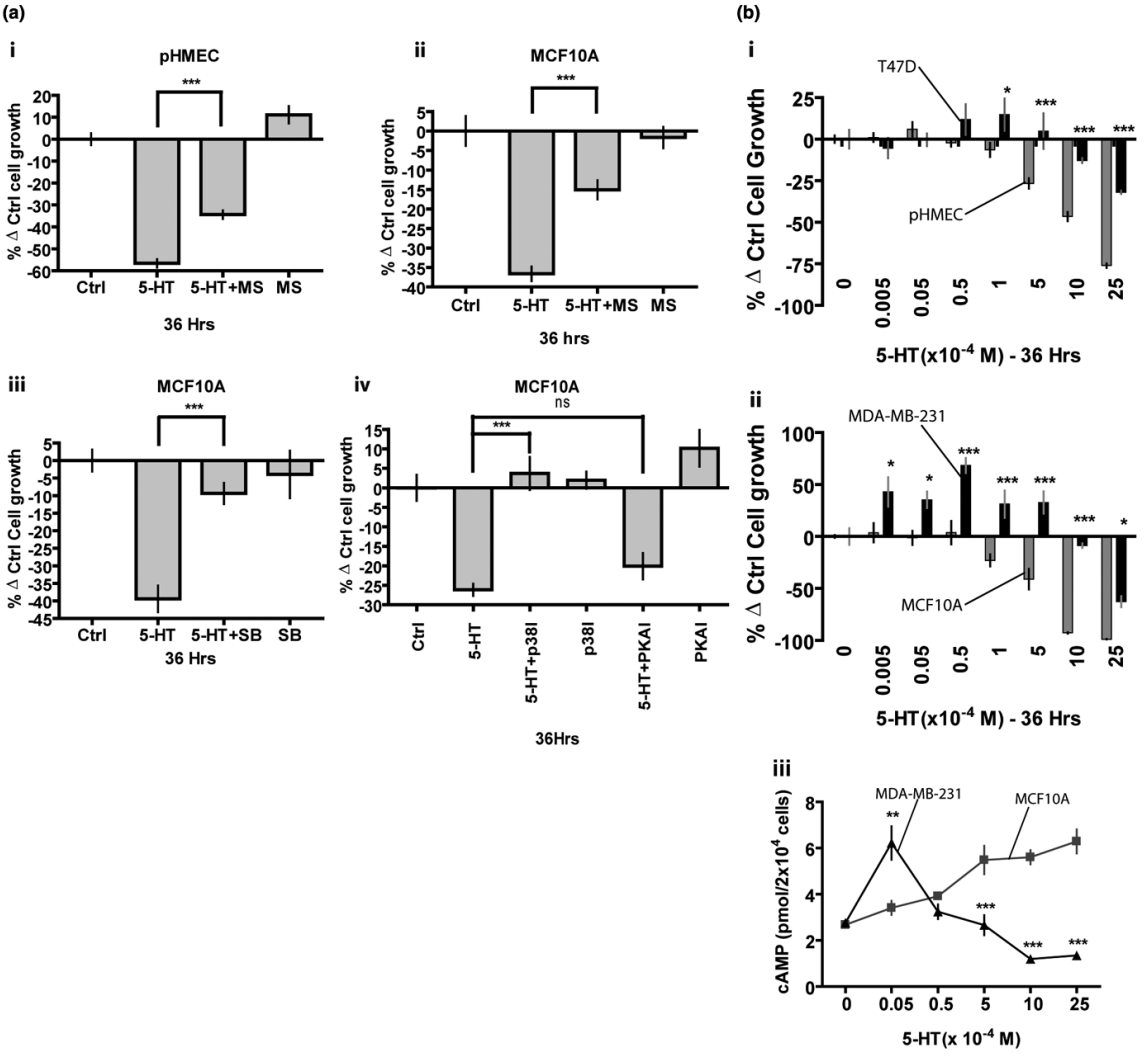
Growth inhibition by 5-HT in non-transformed human mammary epithelial cells is via 5-HT_7 _recepto, r and breast cancer cells are refractory to growth inhibition by 5-HT. **(Ai-iv) **Proliferation assay (MTT) for detecting growth of primary human mammary epithelial cells (pHMECs) **(Ai) **and MCF10A (Aii-iv) after a period of 36 h following indicated treatments. Graphs are plotted as percent change from control (Ctrl - untreated) group. Error bars represent ± SEM. ***P < 0.001 (one-way ANOVA). **(Ai and Aii)**, depict indicated cells treated with 5-HT (7.5 × 10^-4 ^M) in presence or absence of broad-spectrum 5-HT receptor antagonist methysergide (MS) (60 μM). **(Aiii) **depicts MCF10A cells treated with 5-HT (7.5 × 10^-4 ^M) in presence or absence of specific 5-HT_7 _receptor antagonist SB 269970 (SB) (80 μM). (Aiv) depicts MCF10A cells treated with 5-HT (7.5 × 10^-4 ^M) in presence or absence of specific inhibitors of signaling proteins p38 MAPK (SB203580 [p38I]) (20 μM) and PKA (H89 [PKAI]) (10 μM). **(Bi-ii) **depict a proliferation assay (MTT-viable count) for detecting cell growth after 36 h of treatment with indicated 5-HT concentrations. Graphs are plotted as percent change from respective controls (untreated group). Error bars represent ± SEM. *P < 0.05, **P < 0.01, ***P < 0.001 (Two-way ANOVA). **(Bi) **depicts comparison of pHMECs (grey bars) and T47D (black bars) cells response to indicated 5-HT concentrations. **(Bii) **depicts comparison of MCF10A (grey bars) and MDA-MB-231 (black bars) cells response to indicated 5-HT concentrations. **(Biii) **depicts measurements of intracellular cAMP accumulation in MCF10A (grey squares and line) and MDA-MB-231 (black triangle and line) cells in response to 5-HT stimulation as indicated. Error bars in Biii represent ± SEM. ** *P *< 0.01, *** *P *< 0.001 (Two way ANOVA).

Because breast cancer cells were resistant to 5-HT-induced apoptosis, we decided to explore its impact on proliferation in these cells. Treatment with 5-HT for 36 h (in serum-containing medium) resulted in significant inhibition of proliferation in both pHMECs (approximately 55%) and MCF10A (approximately 37%), compared to untreated controls (Figure 6Ai and 6ii). This observation was verified by a standard trypan blue assay under similar experimental conditions [see Figure S5 in Additional data file 1]. Methysergide (MS), a broad spectrum 5-HT receptor antagonist, attenuated growth inhibition by 5-HT in pHMECs and MCF10A (Figure 6Ai and 6ii). Our previous study showed that mammary epithelial cells express the 5-HT_7 _receptor [6] and stimulation of this receptor results in cyclic AMP (cAMP)-mediated activation of both protein kinase A (PKA) and p38 mitogen activated protein kinase (p38 MAPK) [7]. Treatment of MCF10A cells with a 5-HT_7 _antagonist (SB269970 [SB]) resulted in near complete extinction of 5-HT inhibition of proliferation (Figure 6Aiii). In addition, specific inhibition of p38 MAPK blocked the inhibition of proliferation (Figure 6Aiv), however PKA inhibition had no significant effect on proliferation. These data suggest that 5-HT_7_-mediated p38 MAPK activation may, in part, be responsible for growth inhibitory actions of 5-HT in mammary epithelial cells.

### Breast cancer cells are refractory to the growth inhibitory response to 5-HT

It seemed paradoxical that breast cancer cell lines had an increased capacity for 5-HT biosynthesis (Figure 1), while 5-HT acted as a growth-inhibitor in mammary epithelial cells (Figure 6A). The juxtaposition of these findings suggested that the breast cancer cells might be resistant to growth inhibition by 5-HT. Hence, we tested the effect of 5-HT on proliferation of breast cancer cells. Confirming the previous result, 5-HT, in a concentration dependent manner, inhibited proliferation of mammary epithelial cells (pHMECs and MCF10A) (Figure 6B). However, breast cancer cells (T47D and MDA-MB-231) responded differently to 5-HT. The T47D cells were resistant to 5-HT, which required five- to ten-fold higher concentrations to show growth inhibition (Figure 6Bi). However, MDA-MB-231 cells were not only resistant to growth inhibition, but also, were significantly stimulated by 5-HT at low concentrations (Figure 6Aii). The altered response of MDA-MB-231 cells to 5-HT was seen in spite of the presence of strong expression of the HTR7 (Figure 4A and 4Bvi). To determine whether differences in cell death could account for the effects of 5-HT on proliferation, we measured lactate dehydrogenase (LDH) levels in the media, which is indicative of lysed/dead cells. In both the MCF10A and MDA-MB-231 cells, the LDH levels in the media remained unchanged by 36 h after 5-HT treatment [see Figure S6 in Additional data file 1]. At later time points (72 h, see Figure 5), there was substantial apoptosis in the untransformed (i.e., MCF10A) cells and pHMECs, which implied that these cells underwent growth arrest early, followed by apoptosis if 5-HT signaling was sustained.

### Altered 5-HT_7 _signaling in human breast cancer cells

Given that growth inhibition by 5-HT occurs through the 5-HT_7 _receptor, resistance to growth inhibition in breast cancer cells could be explained by: 1] a loss of 5-HT_7 _receptor expression (Figure 4A and 4Bvi), 2] expression of 5-HT receptors that counteract 5-HT_7 _(that is, G_i_-coupled), (Figure 4A, Bii and 6Biii) or 3] changes in signaling downstream of 5-HT_7_; and these possibilities are not mutually exclusive. Expression of HTR7 in MDA-MB-231 cells was greater that in pHMEC and MCF10A, yet MDA-MB-231 cells showed significant differences in their response to 5-HT (Figure 6Bii). Therefore, we sought to determine whether G_s_-coupled signaling, which is downstream of 5-HT_7_, was altered in MDA-MB-231. As previously described [6], MCF10A cells showed a 5-HT concentration-dependent increase in cAMP accumulation (Figure 6Biii). In MDA-MB-231 cells, the maximum cAMP activation was observed at low concentrations of 5-HT, followed by a progressive decline in cAMP to levels below that of controls. Peak cAMP accumulation was identical in both cells (Figure 6Biii). These data imply altered signaling events associated with 5-HT_7_.

## Discussion

Regulation of mammary epithelium involves processes such as epithelial proliferation, invasion of stroma and epithelial regression accompanied by extracellular matrix remodeling. Dysregulation of such epithelial regulatory mechanisms are critically involved in the progression of breast cancers [17,18,42,44-46]. The series of studies reported here were undertaken to establish whether there are functionally-relevant associations between local mammary 5-HT signaling and breast cancers. The simple expedient of identifying serotonergic elements by profiling of either mutations or gene expression was not applicable to this problem because of the complexity of the 5-HT receptor systems. This receptor complexity, and the multiple mechanisms by which the ligand concentration is regulated, make it possible for either normal or cancer cells to evolve completely different sets of signaling interactions to achieve common ends. This characteristic amounts to *convergent evolution *of signaling among cancers, and it required us to use a hypothesis-driven set of approaches, combined with hypothesis-driven data mining.

In our previous studies, we showed that the mammary epithelium expresses the TPH1 gene, which is induced during pregnancy, lactation and milk stasis at the onset of involution [5]. Here we have shown by immunostaining that TPH1 is present primarily in the epithelial tissue of mammary glands. An early finding that piqued our interest in the 5-HT system in human breast cancer was the observation that representative breast cancer cell lines showed significantly elevated TPH1 transcript and protein levels. These results were confirmed by staining for TPH1 protein, which showed uniform and elevated TPH1 expression among cancer cell lines. The shift of TPH1 gene expression from the normal tightly-regulated *in vivo *pattern to a state of homogeneous overexpression in breast cancer cells was reminiscent of the general up-regulation of ER in estrogen-sensitive breast cancer cells [47,48]. Whereas ER and PR expression are positively correlated in breast cancers an interesting observation was no effect of PR status on TPH1 levels. However, it has been reported that PR can be independently regulated in breast cancers [49,50]. Hence, it is likely that TPH1 is regulated by ER independent of PR status. Since TPH1 is rate limiting for 5-HT synthesis in mammary epithelial cells, as in other systems [6], breast cancer cells synthesize excess 5-HT, which they may use to support their growth advantages.

To gain insights into the nature of the 5-HT system in human breast tumors, we used tissue microarray and data-mining approaches. Increased TPH1 expression in cancer cell lines was confirmed on the microarray specimens. An important insight was the observation that there was not a simple linear association between TPH1 expression and cancer stage. Although TPH1 expression was suppressed in primary tumors at early stages, it was increased later in progression. Correspondingly, all of the tissue microarray specimens were harvested from the primary tumor sites of cancers at different stages, but the breast cancer cell lines, which expressed elevated TPH1, were all established from metastasized cells. Therefore, the rebound of TPH1 expression associated with progression may result in metastatic cells that express the highest levels of TPH1.

Carcinogenesis is a complex multistep process, occurring hand-in-hand with metastatic progression. For a normal epithelial cell, there are many internal and external checks and balances that guard against transformation and progression, including cell senescence, apoptosis, appropriate stress responses, and dependence on growth factors and differentiating agents [3,51]. The growth inhibitory effect of 5-HT in the non-transformed mammary epithelium is mediated, in part, through 5-HT_7 _receptor signaling. The growth suppression response to 5-HT is supported by our previous observations in TPH1-/- mice, which showed accelerated mammary growth upon prolactin stimulation, and impaired regression during milk stasis [5].

There was a wide variety of changes in 5-HT receptor expression among the cancer specimens and database results. The convergent nature of cancer evolution, in which there are several routes for cancer cells to take while they accumulate advantageous physiological alterations, provides a context for understanding the diversity of these changes. Down-regulation of 5-HT_7 _(G_s_-coupled), which mediates growth inhibition in untransformed cells, is one route to gain a growth advantage. This particular alteration was seen in MCF7 and T47D cells, and in ER negative and p53-wildtype tumor specimens. Other routes to achieve growth advantage include a) suppression of 5-HT_7 _action through induction of G_i_-coupled receptors, such as 5-HT_1E _and _1F_; b) induction of growth stimulatory 5-HT_2C _(G_q/11_-coupled) [52,53] (observed in MCF7, T47D and in human breast tumors); and c) downregulation of 5-HT synthesis itself, as observed in early stages of primary tumor growth. An important observation was the identical 5-HT receptor expression pattern in both examples of untransformed cells (MCF10A and pHMECs). This indicates that in untransformed mammary epithelial cells, 5-HT receptor expression is under tight control, whereas the control of 5-HT receptor expression was aberrant in all breast cancer cells. Sonier *et al*., [54] previously reported expression of 5-HT_2A _in MCF7 cells, but we did not observe expression of this receptor in either MCF7 or other breast cancer cells. Given the degree to which receptor expression is labile in breast cancer cells, it is conceivable that the subline in their lab is different from the cells we received from ATCC.

One intriguing finding was that 5-HT stimulated higher proliferation rates in MDA-MB-231 cells and promoted obvious morphological changes. Similar phenotypic changes have been correlated with induction of a highly invasive behavior in these cells [42,43].

Proliferative actions of 5-HT are not unprecedented and have been reported to be crucial for liver regeneration [55]. Considering that sustained exposure to 5-HT induces quiescence and apoptosis in untransformed mammary epithelial cells, the response of MDA-MB-231 cells represents a major change in the way these cells interpret the 5-HT signal. This proliferative effect of 5-HT occurs in spite of the continued expression of the 5-HT_7 _receptor, but we have not yet been able to attribute the growth-stimulatory effect to a single 5-HT receptor. The failure of 5-HT to cause growth inhibition and cell death in MDA-MB-231 cells may be attributable to altered downstream 5-HT_7 _signaling. Changes in cAMP dynamics similar to those we observed in MDA-MB-231 cells have been linked to a switch from growth inhibition to growth stimulation in other cells [56-61].

Although some of the normal physiological actions mediated by 5-HT_7 _such as anti-proliferation and pro-apoptosis would inhibit tumor progression, it is not a simple matter that 5-HT_7 _is uniformly tumor-suppressing. Other actions mediated by 5-HT_7 _can enhance tumor progression. Among these is the disruption of cell-to-cell junctions [6,7], which provides an important physiological advantage during invasion and metastasis. Tight junctions have been shown to restrain tumorgenesis, and disrupting tight junctions and other static cell junctions is imperative for tumor growth and invasion [62,62-67].

Although 5-HT signaling can expose cancer cells to a mixture of positive and negative consequences, cancer cells cannot simplify their environment by avoiding 5-HT signaling completely. Apart from the fact of endogenous epithelial 5-HT biosynthesis, cancer cells receive 5-HT signaling from at least three outside sources: neighboring normal breast tissue, normal blood circulation, and hemostatic plaques within tumors (platelet activation and 5-HT release - an essential part of stabilizing the tumor vasculature) [68]. Consequently, there are advantages for breast cancer cells to be gained from altering, rather than avoiding, 5-HT signaling. Presumably, these advantages explain why TPH1 expression does not simply continue to decline to zero in advanced cancers, and is commonly elevated in the most highly-transformed cancer cells. Analogous nonlinear associations with breast cancer progression have been reported in the TGF-β system, which is also generally cytostatic in normal cells but advantageous in advanced cancers [69,70].

While the epithelial cells of breast cancer may be important targets of 5-HT, it is also likely that stromal elements, including connective tissue, adipose, infiltrating blood cells, and vascular elements, are regulated by 5-HT synthesized in breast cancers. The discrepancy between the 5-HT receptor expression profiles of primary mammary epithelial cells (5-HT_1D,2B,3 _and _7_) and in normal whole gland tissue (5-HT_1B,1D,1F_, _2A_, _2B,3,5A _and _7_, Oncomine database) suggests that a subset of 5-HT receptors (5-HT_1F,2A,5A_) are expressed in non-epithelial elements (connective tissue, myopepithelium, blood cells, adipose tissue, vasculature). Some of these extra-epithelial tissues are well known targets of 5-HT (blood vessels and adipose), and others, such as the connective tissue stroma, have not yet been studied. A new study [71] has shown that 5-HT is an important physiological suppressor of osteoblast growth and bone mineralization. Therefore, bone, to which breast cancers preferentially metastasize, may use breast cell-derived 5-HT as an important extra-epithelial target tissue.

**Figure 7:**
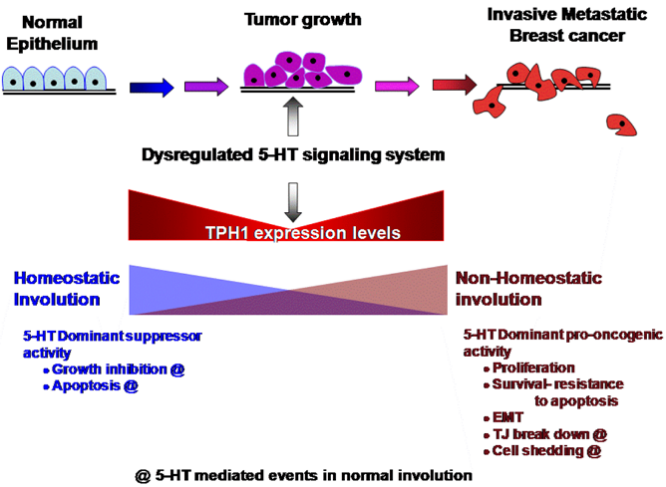
Model of a working hypothesis for 5-HT influences on breast cancer progression. 5-HT is a tumor-suppressing signal in non-transformed breast cells and early stage breast cancers, so early tumor growth relies on reductions in TPH1 expression. During tumor progression, cells acquire genetic or epigenetic alterations in 5-HT signaling which then make them resistant to suppressive 5-HT actions and favor tumor-promoting actions (*e.g*., dynamic cell junctions and cell shedding), as well as acquisition of new receptors and functions (*e.g*., stimulated proliferation and epithelial-mesenchymal transition). The figure is inspired by the image in [69].

Our previous [5-7] and current results are summarized (Figure 7) as follows: a) physiological responses to 5-HT include some that are tumor-suppressing (growth inhibition and apoptosis) and others that are tumor-promoting (junctional breakdown, cell shedding) and because of the growth suppression actions, early tumor growth relies on reductions in TPH1 expression and 5-HT synthesis, and b) as the tumors progress, cells acquire alterations in the 5-HT signal, thus developing resistance to the tumor-suppressive aspects of 5-HT (growth inhibition and apoptosis), while retaining and favoring tumor-promoting actions. These effects are further enhanced by increasing TPH1 levels and acquisition of new tumor promoting-activities (cell proliferation, survival, epithelial-mesenchymal transition phenotype). Fully optimized 5-HT signaling in cancers results in tumors in which 5-HT is used to promote growth, invasiveness, angiogenesis and other patho-physiological effects. While this understanding may need many refinements, it is further supported by the overall trend toward increased complexity of 5-HT receptor expression in association with aggressive tumors, which suggests beneficial influences of 5-HT on tumor progression.

Importantly, we believe the implications of understanding 5-HT physiology extend beyond breast cancers. This is based on reports indicating important roles of 5-HT in the cancers of skin, gut, brain, lung, prostate, liver, and pancreas [20-23,27,28]. Duct systems that are lined by epithelia and require homeostatic regulation represents a common denominator among these organ systems. In some of these organs, like breast and liver, 5-HT has been implicated in homeostatic regulation of secretory ducts [19,29,72].

## Conclusions

Although cancers generally are clonal and may rely on self-renewing stem cells over the long term [3,45,73-76], any given tumor must become heterotypic if it is to reach a substantial size and pathophysiologic complexity. The cancer consists of a community of cells that contribute different pathophysiological properties. The heterotypic nature of a cancer is responsible for its ability to adapt to a changing microenvironment during progression through invasiveness and metastasis. Among the different cell types within a cancer, there are presumably differences in the components and functionality of the 5-HT system. Such differences include cells with a greater or lesser ability to synthesize 5-HT, and cells with different complements of receptors. While this heterotypic 5-HT physiology in breast tumors may create a great deal of complexity, it is possible that future studies will identify common features or weak links in the 5-HT system of breast cancers, which can be exploited therapeutically. The inherent druggability of 5-HT systems makes it reasonable to assume that treatments targeting these systems could achieve sufficiently positive therapeutic indices to justify their uses in cancer.

It would be premature to suggest that studies reported here are sufficient to establish the prognostic or therapeutic value of the breast cancer TPH1/5-HT system. However, an early prediction from our studies is that tumors in which TPH1 is significantly downregulated will have not yet gained resistance to 5-HT or reached metastatic stages. Therefore, TPH1 downregulation may indicate a better prognosis. Through further studies on the breast 5-HT system we expect to improve our understating of its role in breast cancer.

## Abbreviations

5-HT: 5-hydroxytryptamine, serotonin; 5-HT_7_: type 7 serotonin receptor protein; ATCC: American Type Culture Collection; BSA: Bovine Serum Albumin; DMEM: Dulbecco's Modified Eagle Medium; EGF: Epidermal Growth Factor; ER: Estrogen Receptor; FBS: Fetal Bovine Serum; HRP: Horseradish peroxidase; HTR: gene or mRNA encoding serotonin receptor; PBS: Phosphate buffered saline; pHMEC: primary Human Mammary Epithelial Cells; PR: Progesterone Receptor; RT-PCR reverse-transcriptase-couple polymerase chain reaction; SERT: serotonin transporter protein; TPH1: tryptophan hydroxylase 1.

## Competing interests

The authors declare that they have no competing interests.

## Authors' contributions

VPP and NDH designed research, VPP performed research, AMM, LLH. and ARB provided analytical and technical support. NDH and VPP wrote the paper. All authors read and approved the manuscript.

## Supplementary Material

Additional file 1 PDF document containing Table S1, primers used for detecting 5-HT receptors; Figure S1, which compares SERT protein levels between non-transformed and breast cancer cells; Figure S2 is TPH1 staining intensity key for scoring tissue microarray sections; Figure S3 represents changes in TPH1 signal in human breast tumors; Figure S4 represents differences in 5-HT receptor isoform gene expression between non-transformed (pHMECs and MCF10A) and breast cancer cells (MCF7, MDA-MB-231 and T47D; Figure S5 depicts 5-HT inhibition of cell growth in non-transformed mammary epithelial cells; and Figure S6 shows that the 5-HT effect on cell proliferation at 36 h is not influenced by cell death. Click here for file

## References

[B1] Polyak K Breast cancer: origins and evolution J Clin Invest 2007 117 3155 3163 10.1172/JCI33295 17975657 PMC2045618

[B2] Polyak K Haviv I Campbell IG Co-evolution of tumor cells and their microenvironment Trends Genet 2009 25 30 38 10.1016/j.tig.2008.10.012 19054589

[B3] Hanahan D Weinberg RA The hallmarks of cancer Cell 2000 100 57 70 10.1016/S0092-8674(00)81683-9 10647931

[B4] Paez-Ribes M Allen E Hudock J Takeda T Okuyama H Vinals F Inoue M Bergers G Hanahan D Casanovas O Antiangiogenic therapy elicits malignant progression of tumors to increased local invasion and distant metastasis Cancer Cell 2009 15 220 231 10.1016/j.ccr.2009.01.027 19249680 PMC2874829

[B5] Matsuda M Imaoka T Vomachka AJ Gudelsky GA Hou Z Mistry M Bailey JP Nieport KM Walther DJ Bader M Horseman ND Serotonin regulates mammary gland development via an autocrine-paracrine loop Dev Cell 2004 6 193 203 10.1016/S1534-5807(04)00022-X 14960274

[B6] Stull MA Pai V Vomachka AJ Marshall AM Jacob GA Horseman ND Mammary gland homeostasis employs serotonergic regulation of epithelial tight junctions Proc Natl Acad Sci USA 2007 104 16708 16713 10.1073/pnas.0708136104 17940054 PMC2034263

[B7] Pai VP Horseman ND Biphasic Regulation of Mammary Epithelial Resistance by Serotonin through Activation of Multiple Pathways J Biol Chem 2008 283 30901 30910 10.1074/jbc.M802476200 18782769 PMC2576527

[B8] Hernandez LL Stiening CM Wheelock JB Baumgard LH Parkhurst AM Collier RJ Evaluation of serotonin as a feedback inhibitor of lactation in the bovine J Dairy Sci 2008 91 1834 1844 10.3168/jds.2007-0766 18420614

[B9] Hadsell DL Parlow AF Torres D George J Olea W Enhancement of maternal lactation performance during prolonged lactation in the mouse by mouse GH and long-R3-IGF-I is linked to changes in mammary signaling and gene expression J Endocrinol 2008 198 61 70 10.1677/JOE-07-0556 18577570

[B10] Park M Kang K Park S Back K Conversion of 5-hydroxytryptophan into serotonin by tryptophan decarboxylase in plants, Escherichia coli, and yeast Biosci Biotechnol Biochem 2008 72 2456 2458 10.1271/bbb.80220 18776677

[B11] Callera G Tostes R Savoia C Muscara MN Touyz RM Vasoactive peptides in cardiovascular (patho)physiology Expert Rev Cardiovasc Ther 2007 5 531 552 10.1586/14779072.5.3.531 17489676

[B12] Mohammad-Zadeh LF Moses L Gwaltney-Brant SM Serotonin: a review J Vet Pharmacol Ther 2008 31 187 199 10.1111/j.1365-2885.2008.00944.x 18471139

[B13] Crowell MD Wessinger SB 5-HT and the brain-gut axis: opportunities for pharmacologic intervention Expert Opin Investig Drugs 2007 16 761 765 10.1517/13543784.16.6.761 17501688

[B14] Dale GL Coated-platelets: an emerging component of the procoagulant response J Thromb Haemost 2005 3 2185 2192 10.1111/j.1538-7836.2005.01274.x 16194197

[B15] Cote F Thevenot E Fligny C Fromes Y Darmon M Ripoche MA Bayard E Hanoun N Saurini F Lechat P Dandolo L Hamon M Mallet J Vodjdani G Disruption of the nonneuronal tph1 gene demonstrates the importance of peripheral serotonin in cardiac function Proc Natl Acad Sci USA 2003 100 13525 13530 10.1073/pnas.2233056100 14597720 PMC263847

[B16] Lesurtel M Soll C Graf R Clavien PA Role of serotonin in the hepato-gastroIntestinal tract: an old molecule for new perspectives Cell Mol Life Sci 2008 65 940 952 10.1007/s00018-007-7377-3 18080089 PMC11131662

[B17] Russo J Russo IH Toward a physiological approach to breast cancer prevention Cancer Epidemiol Biomarkers Prev 1994 3 353 364 8061586

[B18] Mallon E Osin P Nasiri N Blain I Howard B Gusterson B The basic pathology of human breast cancer J Mammary Gland Biol Neoplasia 2000 5 139 163 10.1023/A:1026439204849 11149570

[B19] Marzioni M Glaser S Francis H Marucci L Benedetti A Alvaro D Taffetani S Ueno Y Roskams T Phinizy JL Venter J Fava G Lesage GD Alpini G Autocrine/paracrine regulation of the growth of the biliary tree by the neuroendocrine hormone serotonin Gastroenterology 2005 128 121 137 10.1053/j.gastro.2004.10.002 15633129

[B20] Alpini G Invernizzi P Gaudio E Venter J Kopriva S Bernuzzi F Onori P Franchitto A Coufal M Frampton G Alvaro D Lee SP Marzioni M Benedetti A DeMorrow S Serotonin metabolism is dysregulated in cholangiocarcinoma, which has implications for tumor growth Cancer Res 2008 68 9184 9193 10.1158/0008-5472.CAN-08-2133 19010890 PMC2593938

[B21] Vicentini LM Cattaneo MG Fesce R Evidence for receptor subtype cross-talk in the mitogenic action of serotonin on human small-cell lung carcinoma cells Eur J Pharmacol 1996 318 497 504 10.1016/S0014-2999(96)00812-6 9016944

[B22] Siddiqui EJ Shabbir M Mikhailidis DP Thompson CS Mumtaz FH The role of serotonin (5-hydroxytryptamine1A and 1B) receptors in prostate cancer cell proliferation J Urol 2006 176 1648 1653 10.1016/j.juro.2006.06.087 16952708

[B23] Sreevidya CS Khaskhely NM Fukunaga A Khaskina P Ullrich SE Inhibition of photocarcinogenesis by platelet-activating factor or serotonin receptor antagonists Cancer Res 2008 68 3978 3984 10.1158/0008-5472.CAN-07-6132 18483284 PMC2394717

[B24] Suzuki A Naruse S Kitagawa M Ishiguro H Yoshikawa T Ko SB Yamamoto A Hamada H Hayakawa T 5-Hydroxytryptamine Strongly Inhibits Fluid Secretion in Guinea Pig Pancreatic Duct Cells J Clin Invest 2001 108 749 756 11544281 10.1172/JCI12312 PMC209377

[B25] Nathan JD Liddle RA Neurohormonal control of pancreatic exocrine secretion Curr Opin Gastroenterol 2002 18 536 544 10.1097/00001574-200209000-00003 17033330

[B26] Russo F Vittoria A Neuroendocrine cells in the vestibular glands of the genital tract of cows and pigs Acta Histochem 2006 108 351 355 10.1016/j.acthis.2006.06.004 16997356

[B27] Ogawa T Sugidachi A Tanaka N Fujimoto K Fukushige J Tani Y Asai F Effects of R-102444 and its active metabolite R-96544, selective 5-HT2A receptor antagonists, on experimental acute and chronic pancreatitis: Additional evidence for possible involvement of 5-HT2A receptors in the development of experimental pancreatitis. Eur J Pharmacol 6544 521 156 163 10.1016/j.ejphar.2005.08.033 16183055

[B28] Siddiqui EJ Thompson CS Mikhailidis DP Mumtaz FH The role of serotonin in tumour growth (review) Oncol Rep 2005 14 1593 1597 16273262

[B29] Van Lommel A Pulmonary neuroendocrine cells (PNEC) and neuroepithelial bodies (NEB): chemoreceptors and regulators of lung development Paediatr Respir Rev 2001 2 171 176 10.1053/prrv.2000.0126 12531066

[B30] Ethier SP Summerfelt RM Cundiff KC Asch BB The influence of growth factors on the proliferative potential of normal and primary breast cancer-derived human breast epithelial cells Breast Cancer Res Treat 1991 17 221 230 10.1007/BF01806371 1710154

[B31] The National Cancer Institute-Cooperative Breast Cancer Tissue http://cbctr.nci.nih.gov/

[B32] Huang Y Li X Jiang J Frank SJ Prolactin modulates phosphorylation, signaling and trafficking of epidermal growth factor receptor in human T47D breast cancer cells Oncogene 2006 25 7565 7576 10.1038/sj.onc.1209740 16785991

[B33] Tonner E Barber MC Travers MT Logan A Flint DJ Hormonal control of insulin-like growth factor-binding protein-5 production in the involuting mammary gland of the rat Endocrinology 1997 138 5101 5107 10.1210/en.138.12.5101 9389489

[B34] McCarty KS Jr Miller LS Cox EB Konrath J McCarty KS Sr Estrogen receptor analyses. Correlation of biochemical and immunohistochemical methods using monoclonal antireceptor antibodies Arch Pathol Lab Med 1985 109 716 721 3893381

[B35] Abd El-Rehim DM Ball G Pinder SE Rakha E Paish C Robertson JF Macmillan D Blamey RW Ellis IO High-throughput protein expression analysis using tissue microarray technology of a large well-characterised series identifies biologically distinct classes of breast cancer confirming recent cDNA expression analyses Int J Cancer 2005 116 340 350 10.1002/ijc.21004 15818618

[B36] Rhodes DR Kalyana-Sundaram S Mahavisno V Varambally R Yu J Briggs BB Barrette TR Anstet MJ Kincead-Beal C Kulkarni P Varambally S Ghosh D Chinnaiyan AM Oncomine 3.0: genes, pathways, and networks in a collection of 18,000 cancer gene expression profiles Neoplasia 2007 9 166 180 10.1593/neo.07112 17356713 PMC1813932

[B37] Oncomine database http://www.oncomine.org

[B38] Nilsson T Longmore J Shaw D Pantev E Bard JA Branchek T Edvinsson L Characterisation of 5-HT receptors in human coronary arteries by molecular and pharmacological techniques Eur J Pharmacol 1999 372 49 56 10.1016/S0014-2999(99)00114-4 10374714

[B39] Kato S Kumamoto H Hirano M Akiyama H Kaneko N Expression of 5-HT2A and 5-HT1B receptor mRNA in blood vessels Mol Cell Biochem 1999 199 57 61 10.1023/A:1006999031932 10544952

[B40] Motyl T Gajkowska B Zarzynska J Gajewska M Lamparska-Przybysz M Apoptosis and autophagy in mammary gland remodeling and breast cancer chemotherapy J Physiol Pharmacol 2006 57 Suppl 7 17 32 17228094

[B41] Stein T Salomonis N Gusterson BA Mammary gland involution as a multi-step process J Mammary Gland Biol Neoplasia 2007 12 25 35 10.1007/s10911-007-9035-7 17431797

[B42] Bemis LT Schedin P Reproductive state of rat mammary gland stroma modulates human breast cancer cell migration and invasion Cancer Res 2000 60 3414 3418 10910049

[B43] McDaniel SM Rumer KK Biroc SL Metz RP Singh M Porter W Schedin P Remodeling of the mammary microenvironment after lactation promotes breast tumor cell metastasis Am J Pathol 2006 168 608 620 10.2353/ajpath.2006.050677 16436674 PMC1606507

[B44] Schedin P O'Brien J Rudolph M Stein T Borges V Microenvironment of the involuting mammary gland mediates mammary cancer progression J Mammary Gland Biol Neoplasia 2007 12 71 82 10.1007/s10911-007-9039-3 17318269

[B45] Polyak K Breast cancer: origins and evolution J Clin Invest 2007 117 3155 3163 10.1172/JCI33295 17975657 PMC2045618

[B46] Howard BA Gusterson BA Human breast development J Mammary Gland Biol Neoplasia 2000 5 119 137 10.1023/A:1026487120779 11149569

[B47] Clarke RB Howell A Potten CS Anderson E Dissociation between steroid receptor expression and cell proliferation in the human breast Cancer Res 1997 57 4987 4991 9371488

[B48] Jarzabek K Koda M Kozlowski L Mittre H Sulkowski S Kottler ML Wolczynski S Distinct mRNA, protein expression patterns and distribution of oestrogen receptors alpha and beta in human primary breast cancer: correlation with proliferation marker Ki-67 and clinicopathological factors Eur J Cancer 2005 41 2924 2934 10.1016/j.ejca.2005.09.010 16289616

[B49] Hewitt SC Korach KS Progesterone action and responses in the alphaERKO mouse Steroids 2000 65 551 557 10.1016/S0039-128X(00)00113-6 11108859

[B50] Lange CA Challenges to defining a role for progesterone in breast cancer Steroids 2008 73 914 921 10.1016/j.steroids.2007.12.023 18243264 PMC2481303

[B51] Paez-Ribes M Allen E Hudock J Takeda T Okuyama H Vinals F Inoue M Bergers G Hanahan D Casanovas O Antiangiogenic therapy elicits malignant progression of tumors to increased local invasion and distant metastasis Cancer Cell 2009 15 220 231 10.1016/j.ccr.2009.01.027 19249680 PMC2874829

[B52] Westphal RS Sanders-Bush E Differences in agonist-independent and -dependent 5-hydroxytryptamine2C receptor-mediated cell division Mol Pharmacol 1996 49 474 480 8643087

[B53] De Lucchini S Ori M Nardini M Marracci S Nardi I Expression of 5-HT2B and 5-HT2C receptor genes is associated with proliferative regions of Xenopus developing brain and eye Brain Res Mol Brain Res 2003 115 196 201 10.1016/S0169-328X(03)00173-6 12877990

[B54] Sonier B Arseneault M Lavigne C Ouellette RJ Vaillancourt C The 5-HT2A serotoninergic receptor is expressed in the MCF-7 human breast cancer cell line and reveals a mitogenic effect of serotonin Biochem Biophys Res Commun 2006 343 1053 1059 10.1016/j.bbrc.2006.03.080 16580628

[B55] Lesurtel M Graf R Aleil B Walther DJ Tian Y Jochum W Gachet C Bader M Clavien PA Platelet-derived serotonin mediates liver regeneration Science 2006 312 104 107 10.1126/science.1123842 16601191

[B56] Bombik BM Burger MM c-AMP and the cell cycle: inhibition of growth stimulation Exp Cell Res 1973 80 88 94 10.1016/0014-4827(73)90278-4 4361349

[B57] Burger MM Bombik BM Breckenridge BM Sheppard JR Growth control and cyclic alterations of cyclic AMP in the cell cycle Nat New Biol 1972 239 161 163 10.1038/239161a0 4349672

[B58] Sheppard JR Difference in the cyclic adenosine 3',5'-monophosphate levels in normal and transformed cells Nat New Biol 1972 236 14 16 4336394 10.1038/newbio236014a0

[B59] Ryan WL Heidrick ML Inhibition of cell growth in vitro by adenosine 3',5'-monophosphate Science 1968 162 1484 1485 10.1126/science.162.3861.1484 4301778

[B60] Ryan WL Heidrick ML Role of cyclic nucleotides in cancer Adv Cyclic Nucleotide Res 1974 4 81 116 4369152

[B61] Heidrick ML Ryan WL Adenosine 3',5'-cyclic monophosphate and contact inhibition Cancer Res 1971 31 1313 1315 4329885

[B62] Birchmeier W E-cadherin as a tumor (invasion) suppressor gene Bioessays 1995 17 97 99 10.1002/bies.950170203 7748170

[B63] Itoh M Bissell MJ The organization of tight junctions in epithelia: implications for mammary gland biology and breast tumorigenesis J Mammary Gland Biol Neoplasia 2003 8 449 462 10.1023/B:JOMG.0000017431.45314.07 14985640 PMC2933220

[B64] Hoevel T Macek R Mundigl O Swisshelm K Kubbies M Expression and targeting of the tight junction protein CLDN1 in CLDN1-negative human breast tumor cells J Cell Physiol 2002 191 60 68 10.1002/jcp.10076 11920682

[B65] Swisshelm K Machl A Planitzer S Robertson R Kubbies M Hosier S SEMP1, a senescence-associated cDNA isolated from human mammary epithelial cells, is a member of an epithelial membrane protein superfamily Gene 1999 226 285 295 10.1016/S0378-1119(98)00553-8 9931503

[B66] Hoover KB Liao SY Bryant PJ Loss of the tight junction MAGUK ZO-1 in breast cancer: relationship to glandular differentiation and loss of heterozygosity Am J Pathol 1998 153 1767 1773 9846967 10.1016/S0002-9440(10)65691-X PMC1866327

[B67] Chlenski A Ketels KV Korovaitseva GI Talamonti MS Oyasu R Scarpelli DG Organization and expression of the human zo-2 gene (tjp-2) in normal and neoplastic tissues Biochim Biophys Acta 2000 1493 319 324 11018256 10.1016/s0167-4781(00)00185-8

[B68] Ho-Tin-Noe B Goerge T Cifuni SM Duerschmied D Wagner DD Platelet granule secretion continuously prevents intratumor hemorrhage Cancer Res 2008 68 6851 6858 10.1158/0008-5472.CAN-08-0718 18701510 PMC2547489

[B69] Roberts AB Wakefield LM The two faces of transforming growth factor beta in carcinogenesis Proc Natl Acad Sci USA 2003 100 8621 8623 10.1073/pnas.1633291100 12861075 PMC166359

[B70] Bakin AV Rinehart C Tomlinson AK Arteaga CL p38 mitogen-activated protein kinase is required for TGFbeta-mediated fibroblastic transdifferentiation and cell migration J Cell Sci 2002 115 3193 3206 12118074 10.1242/jcs.115.15.3193

[B71] Yadav VK Ryu JH Suda N Tanaka KF Gingrich JA Schutz G Glorieux FH Chiang CY Zajac JD Insogna KL Mann JJ Hen R Ducy P Karsenty G Lrp5 controls bone formation by inhibiting serotonin synthesis in the duodenum Cell 2008 135 825 837 10.1016/j.cell.2008.09.059 19041748 PMC2614332

[B72] Bayer H Muller T Myrtek D Sorichter S Ziegenhagen M Norgauer J Zissel G Idzko M Serotoninergic receptors on human airway epithelial cells Am J Respir Cell Mol Biol 2007 36 85 93 10.1165/rcmb.2006-0151OC 16873768

[B73] Polyak K Is breast tumor progression really linear? Clin Cancer Res 2008 14 339 341 10.1158/1078-0432.CCR-07-2188 18223205

[B74] Nowell PC The clonal evolution of tumor cell populations Science 1976 194 23 28 10.1126/science.959840 959840

[B75] Ginestier C Wicha MS Mammary stem cell number as a determinate of breast cancer risk Breast Cancer Res 2007 9 109 10.1186/bcr1741 17688678 PMC2206714

[B76] Chang CC Recent translational research: stem cells as the roots of breast cancer Breast Cancer Res 2006 8 103 10.1186/bcr1385 16524453 PMC1413993

[B77] Richardson AL Wang ZC De Nicolo A Lu X Brown M Miron A Liao X Iglehart JD Livingston DM Ganesan S X chromosomal abnormalities in basal-like human breast cancer Cancer Cell 2006 9 121 132 10.1016/j.ccr.2006.01.013 16473279

[B78] Expression Project for Oncology - Breast Samples http://www.ncbi.nlm.nih.gov/geo/query/acc.cgi?acc=GSE2109

[B79] Yu K Ganesan K Miller LD Tan P A modular analysis of breast cancer reveals a novel low-grade molecular signature in estrogen receptor-positive tumors Clin Cancer Res 2006 12 3288 3296 10.1158/1078-0432.CCR-05-1530 16740749

[B80] Ivshina AV George J Senko O Mow B Putti TC Smeds J Lindahl T Pawitan Y Hall P Nordgren H Wong JE Liu ET Bergh J Kuznetsov VA Miller LD Genetic reclassification of histologic grade delineates new clinical subtypes of breast cancer Cancer Res 2006 66 10292 10301 10.1158/0008-5472.CAN-05-4414 17079448

[B81] Miller WR Larionov AA Renshaw L Anderson TJ White S Murray J Murray E Hampton G Walker JR Ho S Krause A Evans DB Dixon JM Changes in breast cancer transcriptional profiles after treatment with the aromatase inhibitor, letrozole Pharmacogenet Genomics 2007 17 813 826 10.1097/FPC.0b013e32820b853a 17885619

[B82] Ginestier C Cervera N Finetti P Esteyries S Esterni B Adelaide J Xerri L Viens P Jacquemier J Charafe-Jauffret E Chaffanet M Birnbaum D Bertucci F Prognosis and gene expression profiling of 20q13-amplified breast cancers Clin Cancer Res 2006 12 4533 4544 10.1158/1078-0432.CCR-05-2339 16899599

[B83] van 't Veer LJ Dai H Vijver MJ van de He YD Hart AA Mao M Peterse HL Kooy K van der Marton MJ Witteveen AT Schreiber GJ Kerkhoven RM Roberts C Linsley PS Bernards R Friend SH Gene expression profiling predicts clinical outcome of breast cancer Nature 2002 415 530 536 10.1038/415530a 11823860

[B84] Chin SF Teschendorff AE Marioni JC Wang Y Barbosa-Morais NL Thorne NP Costa JL Pinder SE Wiel MA van de Green AR Ellis IO Porter PL Tavare S Brenton JD Ylstra B Caldas C High-resolution aCGH and expression profiling identifies a novel genomic subtype of ER negative breast cancer Genome Biol 2007 8 R215 10.1186/gb-2007-8-10-r215 17925008 PMC2246289

[B85] Minn AJ Gupta GP Siegel PM Bos PD Shu W Giri DD Viale A Olshen AB Gerald WL Massague J Genes that mediate breast cancer metastasis to lung Nature 2005 436 518 524 10.1038/nature03799 16049480 PMC1283098

[B86] Hess KR Anderson K Symmans WF Valero V Ibrahim N Mejia JA Booser D Theriault RL Buzdar AU Dempsey PJ Rouzier R Sneige N Ross JS Vidaurre T Gomez HL Hortobagyi GN Pusztai L Pharmacogenomic predictor of sensitivity to preoperative chemotherapy with paclitaxel and fluorouracil, doxorubicin, and cyclophosphamide in breast cancer J Clin Oncol 2006 24 4236 4244 10.1200/JCO.2006.05.6861 16896004

[B87] Sotiriou C Wirapati P Loi S Harris A Fox S Smeds J Nordgren H Farmer P Praz V Haibe-Kains B Desmedt C Larsimont D Cardoso F Peterse H Nuyten D Buyse M Vijver MJ Van de Bergh J Piccart M Delorenzi M Gene expression profiling in breast cancer: understanding the molecular basis of histologic grade to improve prognosis J Natl Cancer Inst 2006 98 262 272 16478745 10.1093/jnci/djj052

[B88] Wang Y Klijn JG Zhang Y Sieuwerts AM Look MP Yang F Talantov D Timmermans M Meijer-van Gelder ME Yu J Jatkoe T Berns EM Atkins D Foekens JA Gene-expression profiles to predict distant metastasis of lymph-node-negative primary breast cancer Lancet 2005 365 671 679 15721472 10.1016/S0140-6736(05)17947-1

[B89] Desmedt C Piette F Loi S Wang Y Lallemand F Haibe-Kains B Viale G Delorenzi M Zhang Y d'Assignies MS Bergh J Lidereau R Ellis P Harris AL Klijn JG Foekens JA Cardoso F Piccart MJ Buyse M Sotiriou C TRANSBIG Consortium Strong time dependence of the 76-gene prognostic signature for node-negative breast cancer patients in the TRANSBIG multicenter independent validation series Clin Cancer Res 2007 13 3207 3214 10.1158/1078-0432.CCR-06-2765 17545524

[B90] Farmer P Bonnefoi H Becette V Tubiana-Hulin M Fumoleau P Larsimont D Macgrogan G Bergh J Cameron D Goldstein D Duss S Nicoulaz AL Brisken C Fiche M Delorenzi M Iggo R Identification of molecular apocrine breast tumours by microarray analysis Oncogene 2005 24 4660 4671 10.1038/sj.onc.1208561 15897907

[B91] Vijver MJ van de He YD van't Veer LJ Dai H Hart AA Voskuil DW Schreiber GJ Peterse JL Roberts C Marton MJ Parrish M Atsma D Witteveen A Glas A Delahaye L Velde T van der Bartelink H Rodenhuis S Rutgers ET Friend SH Bernards R A gene-expression signature as a predictor of survival in breast cancer N Engl J Med 2002 347 1999 2009 10.1056/NEJMoa021967 12490681

[B92] Bild AH Yao G Chang JT Wang Q Potti A Chasse D Joshi MB Harpole D Lancaster JM Berchuck A Olson JA Jr Marks JR Dressman HK West M Nevins JR Oncogenic pathway signatures in human cancers as a guide to targeted therapies Nature 2006 439 353 357 10.1038/nature04296 16273092

