# Supplementary material for: Altered serotonin physiology in human breast cancers favors paradoxical growth and cell survival
Source: Breast Cancer Res. 2009 Nov 10;11(6):R81. doi: 10.1186/bcr2448 (PMC2815543; doi:10.1186/bcr2448)
Supplement: Additional file 1 — PDF document containing Table S1, primers used for detecting 5-HT receptors; Figure S1, which compares SERT protein levels between non-transformed and breast cancer cells; Figure S2 is TPH1 staining intensity key for scoring tissue microarray sections; Figure S3 represents changes in TPH1 signal in human breast tumors; Figure S4 represents differences in 5-HT receptor isoform gene expression between non-transformed (pHMECs and MCF10A) and breast cancer cells (MCF7, MDA-MB-231 and T47D; Figure S5 depicts 5-HT inhibition of cell growth in non-transformed mammary epithelial cells; and Figure S6 shows that the 5-HT effect on cell proliferation at 36 h is not influenced by cell death. [file bcr2448-S1.PDF]

Table S1

Receptor	Left Primer sequence	Right Primer sequence
5-HT1A	GAGCTTTCTACATCCCGCTG	CAGAACAAGAGCCACGATGA
5-HT1B	AAAGTACTGCTGGTTATGCTATTGG	AGATAGAGATGGAGAAGACCCACAC
5-HT1D	CTGGACAGGTACTGGGCAAT	AAGCGCTTCCCATAGAGTGA
5-HT1E	TACCACGCGGCCAAGAGCCTTTACCA	TGGTGCTAGAGATCTGCTGACGTTC
5-HT1F	CCTTCAGCATTGTGTATATTGTGAG	GATGTGGAAACTGATTTAGTGCTTT
5-HT2A	GTGATGTGGTGCCCTTTCTT	GAGACAACCATTGAGCAGTCA
5-HT2B	GCCTTCTTCACACCTCTTGC	AGGCTCTCTGTTCGTTGGAA
5-HT2C	CCCATTTTTCATTACCAATATTCTG	ATTCTCAACTTGCATCTCTATACCG
5-HT3A	CGGTCTTCCTGATCATCGTT	GCGATTCTCTCCAGAACCAG
5-HT4	TATTACCGCATCTATGTCACAGCTA	AGGCACGTCTAAAAGACTTATTCAA
5-HT5A	GAGTGCTTATTCTCACCTTGCTG	CAGCCTTGTAGATCTTCCAGTACAC
5-HT6	CAACACGTCCAACTTCTTCCT	GAGAGGATGAGCAGGTAGCG
5-HT7abd	CCTTCATCTGTGGCACTTCC	GGTGTGCTTACTGCTGAGAT
5-HT7	ACAGAAGGCTTTTCCAAGCA	ACCCTTCAGAGCACGAGAAA

Figure S1

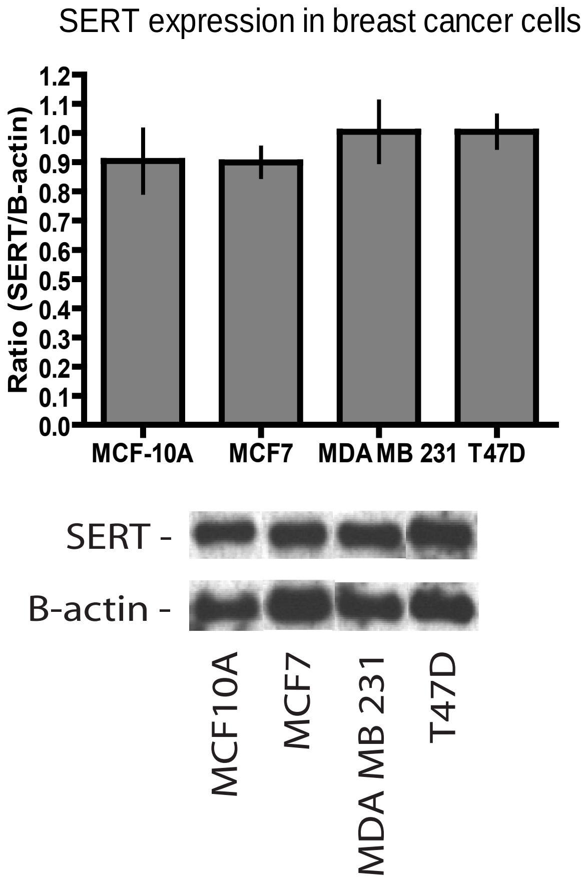
SERT expression in breast cancer cells
Ratio (SERT/B-actin)
1.2
1.1
1.0
0.9
0.8
0.7
0.6
0.5
0.4
0.3
0.2
0.1
0.0
MCF-10A
MCF7
MDA MB 231
T47D
SERT -
B-actin -
MCF10A
MCF7
MDA MB 231
T47D

Figure S2

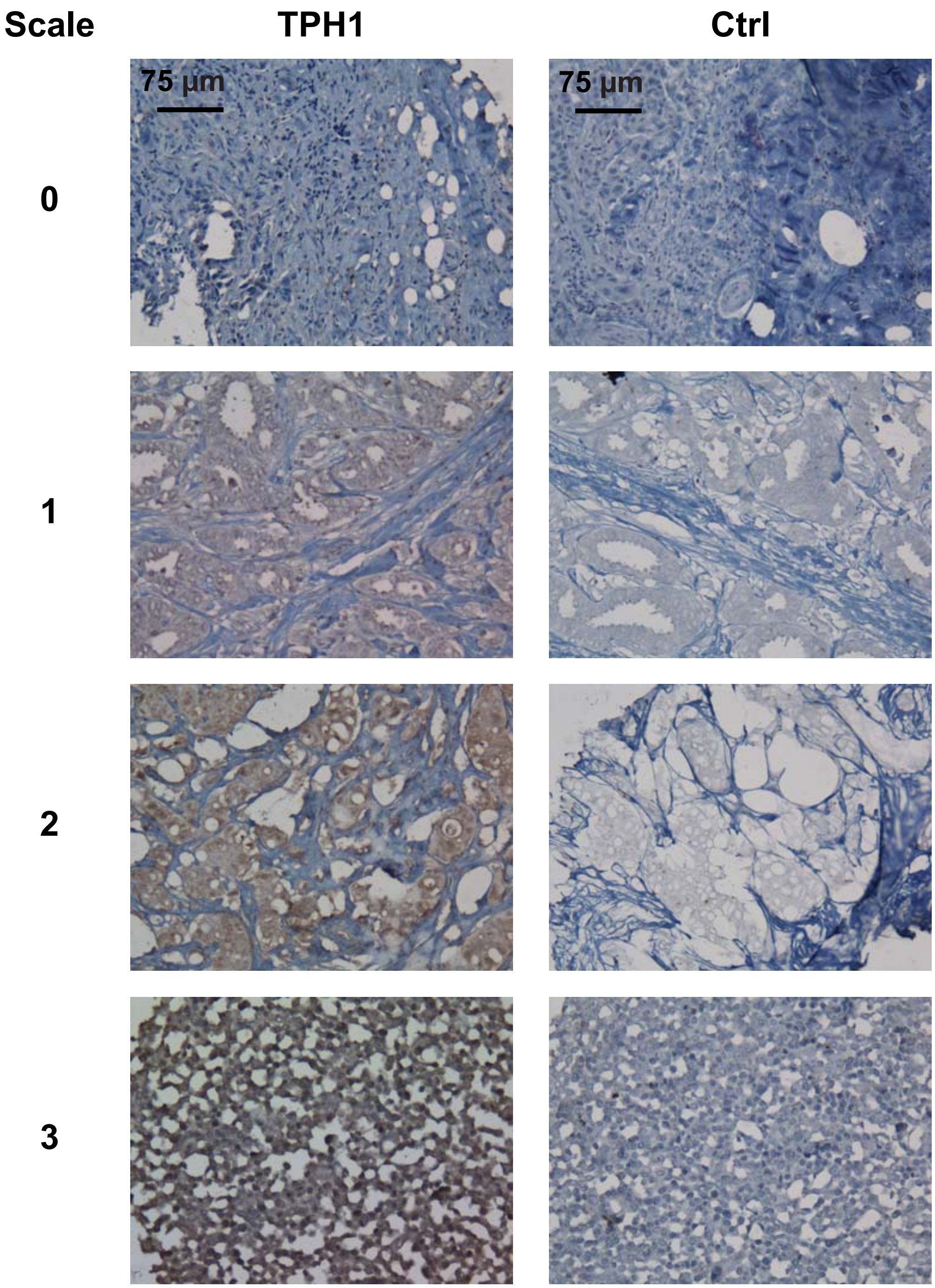

Figure S3

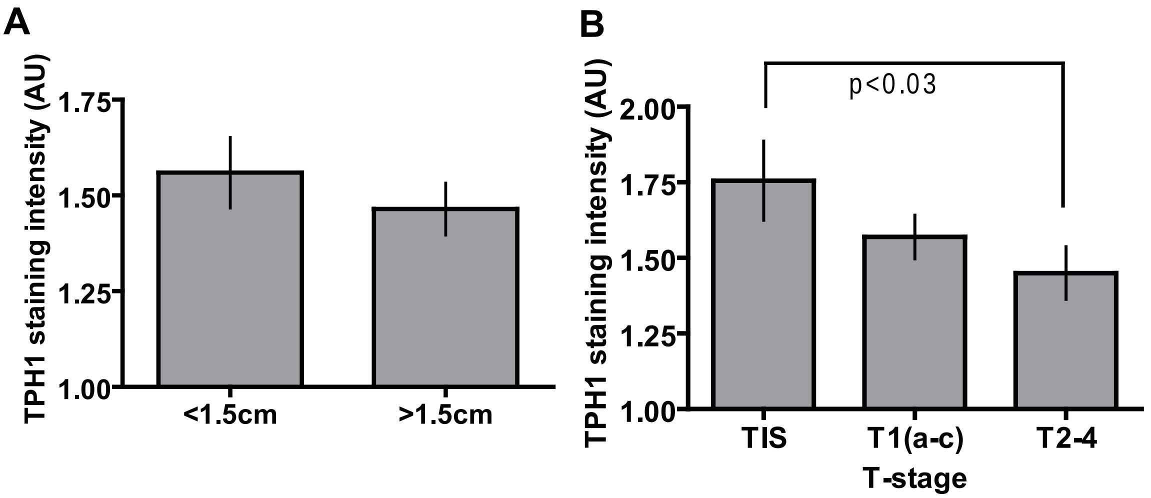
A
TPH1 staining intensity (AU)
1.75
1.50
1.25
1.00
<1.5cm
>1.5cm
B
p<0.03
TPH1 staining intensity (AU)
2.00
1.75
1.50
1.25
1.00
TIS
T1(a-c)
T2-4
T-stage

Figure S4

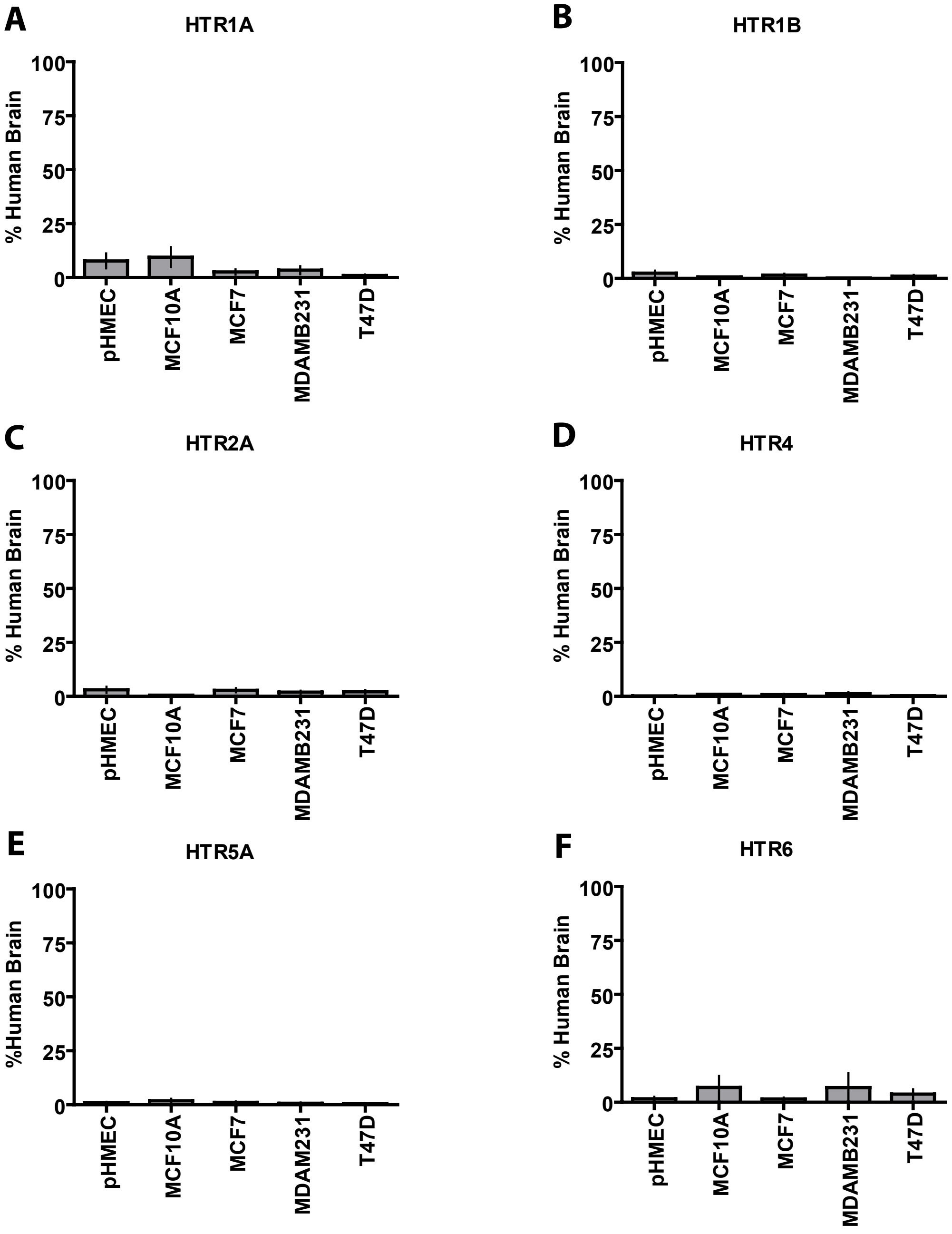

A
HTR1A
B
HTR1B
C
HTR2A
D
HTR4
E
HTR5A
F
HTR6
% Human Brain
%Human Brain
100
75
50
25
0
pHMEC
MCF10A
MCF7
MDAMB231
MDAM231
T47D

Figure S5

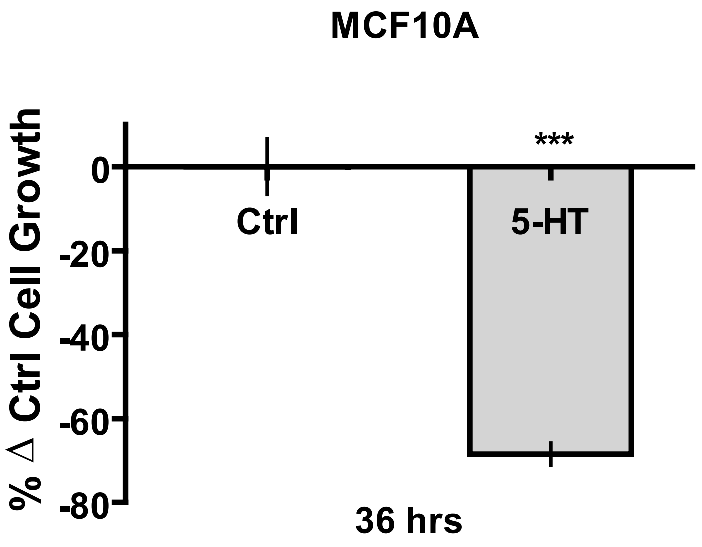

Figure S6

A

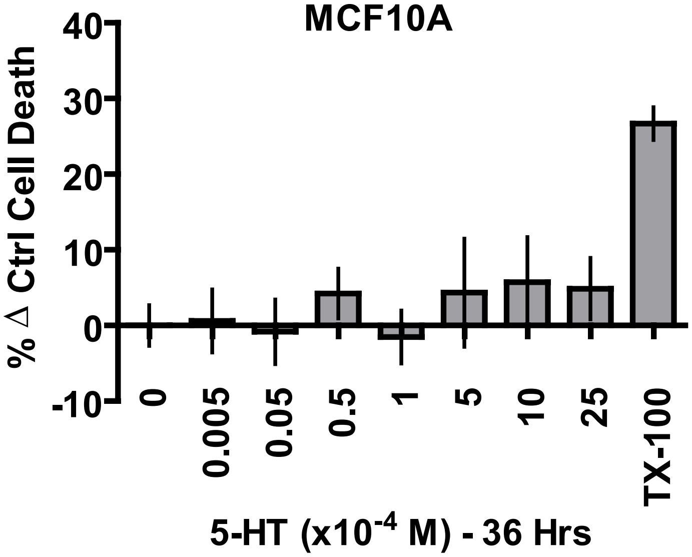
MCF10A
40
30
20
10
0
-10
% Δ Ctrl Cell Death
0
0.005
0.05
0.5
1
5
10
25
TX-100
5-HT (x10^-4 M) - 36 Hrs

B

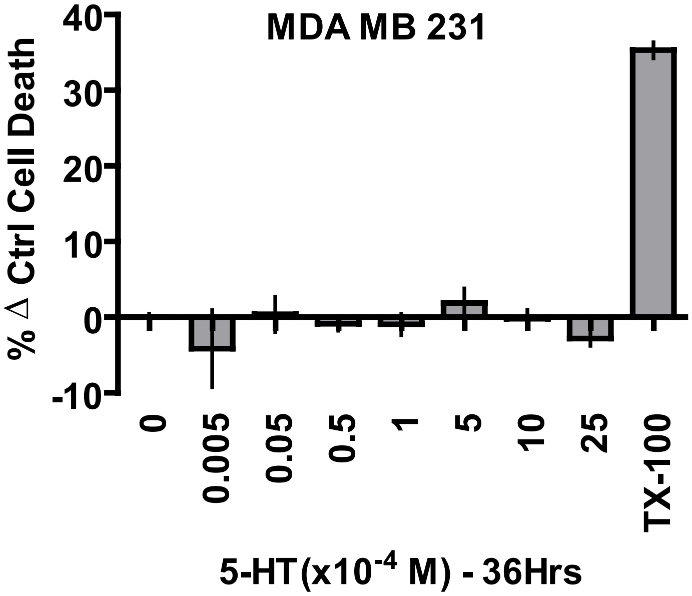
MDA MB 231
40
30
20
10
0
-10
% Δ Ctrl Cell Death
0
0.005
0.05
0.5
1
5
10
25
TX-100
5-HT(x10^-4 M) - 36Hrs
